# Proteome-Based Maternal Plasma and Serum Biomarkers for Preeclampsia: A Systematic Review and Meta-Analysis

**DOI:** 10.3390/life15050776

**Published:** 2025-05-13

**Authors:** Natalia Starodubtseva, Alina Poluektova, Alisa Tokareva, Evgenii Kukaev, Anna Avdeeva, Elena Rimskaya, Zulfiya Khodzayeva

**Affiliations:** 1 V.I. Kulakov National Medical Research Center for Obstetrics, Gynecology and Perinatology, Ministry of Healthcare of Russian Federation, 117997 Moscow, Russia; a_poluektova@oparina4.ru (A.P.); a_tokareva@oparina4.ru (A.T.); e_kukaev@oparina4.ru (E.K.); a_avdeeva@oparina4.ru (A.A.); e_rimskaya@oparina4.ru (E.R.); z_khodzhaeva@oparina4.ru (Z.K.); 2 V.L. Talrose Institute for Energy Problems of Chemical Physics, N.N. Semenov Federal Research Center for Chemical Physics, Russian Academy of Sciences, 119334 Moscow, Russia; 3 Moscow Center for Advanced Studies, 123592 Moscow, Russia; 4 Lebedev Physical Institute, 119991 Moscow, Russia

**Keywords:** proteomics, preeclampsia, plasma, serum, prognosis, diagnostics, biomarkers, pregnancy

## Abstract

Proteomics has emerged as a transformative tool in biomedical research, enabling comprehensive characterization of protein profiles in complex biological systems. In preeclampsia (PE) research, quantitative proteomic analyses of plasma and serum have revealed critical insights into disease mechanisms and potential biomarkers. Through a systematic review of 17 studies (2009–2024), we identified 561 differentially expressed plasma/serum proteins (*p* < 0.05) in PE patients versus healthy controls, with 122 proteins consistently replicated across ≥2 independent studies. Stratified analysis by clinical subtype (early-vs. late-onset PE) demonstrated both concordant and divergent protein expression patterns, reflecting heterogeneity in PE pathophysiology, methodological variations (e.g., sample processing, proteomic platforms), and differences between discovery-phase and targeted validation studies. The trimester-specific biomarker panels proposed here offer a framework for future large-scale, multicenter validation. By integrating advanced proteomic technologies with standardized preanalytical and analytical protocols, these findings advance opportunities for early prediction (first-trimester biomarker signatures); mechanistic insight (complement system involvement); and personalized management (subtype-specific therapeutic targets). This work underscores the potential of proteomics to reshape PE research, from molecular discovery to clinical translation, ultimately improving outcomes for this leading cause of maternal and perinatal morbidity.

## 1. Introduction

Preeclampsia (PE) represents a complex multisystem disorder affecting approximately 8% of pregnancies worldwide, remaining a leading cause of maternal and perinatal mortality [1,2]. The rising global incidence correlates with increasing prevalence of established risk factors including genetic predisposition, advanced maternal age, and preexisting medical conditions such as chronic hypertension, diabetes mellitus, renal disease, and autoimmune disorders [3]. Current diagnostic criteria, as outlined by the UK National Institute for Health and Care Excellence (NICE), require the presence of new-onset hypertension (defined as systolic/diastolic blood pressure ≥ 140/90 mmHg) accompanied by either significant proteinuria (≥300 mg/24 h) or systemic manifestations including oliguria, persistent headache, or right upper quadrant pain [4]. Clinicians typically classify PE based on gestational timing, distinguishing between early-onset (diagnosis before 34 weeks) and late-onset (after 34 weeks) forms, with the former associated with more severe complications and poorer outcomes [5,6]. Additional classification considers delivery timing, with preterm PE requiring delivery before 37 weeks and term PE managed at or beyond 37 weeks gestation.

When undiagnosed or inadequately managed, PE can progress to life-threatening maternal complications including eclampsia, HELLP syndrome (characterized by hemolysis, elevated liver enzymes, and low platelets), and acute kidney injury. Notably, cerebrovascular events such as strokes and cerebral edema account for nearly 40% of PE-related maternal deaths [7]. Cardiovascular research conducted in collaboration with the American Heart Association has demonstrated significant cardiac involvement in early-onset PE, manifesting as impaired myocardial contractility, diastolic dysfunction, ventricular hypertrophy, and pathological remodeling [8,9]. From a fetal perspective, PE presents significant risks, including chronic intrauterine hypoxia and growth restriction, which may predispose offspring to long-term cardiovascular, respiratory, and metabolic disorders. These include hypertension, respiratory distress syndrome, bronchopulmonary dysplasia, obesity, and dysregulation of the renin–angiotensin–aldosterone system. Additionally, children exposed to PE face an elevated risk of neurodevelopmental impairments, ranging from cerebral palsy to intellectual disability and autism spectrum disorder [7]. These widespread consequences underscore the urgent need for improved early detection and diagnostic strategies in modern obstetric practice.

PE has long been termed a “disease of theories” due to persistent uncertainties surrounding its pathogenesis. The prevailing hypothesis centers on inadequate cytotrophoblast invasion during early placentation, resulting in defective spiral artery remodeling. This placental dysfunction initiates a cascade of systemic inflammation and widespread endothelial damage [10]. The consequent vascular abnormalities not only impair uteroplacental perfusion but may also promote atherosclerotic-like changes in maternal vasculature, further aggravating placental ischemia and endothelial injury [10,11,12]. The American College of Obstetricians and Gynecologists (ACOG) recommends low-dose aspirin prophylaxis to mitigate PE risk [13]. Although aspirin reduces preterm PE incidence by 18–48%—primarily by preventing spiral artery thrombosis—its efficacy remains partial [14]. Emerging evidence also underscores the role of systemic angiogenic imbalance, bridging placental pathology to clinical disease manifestations.

Modern investigations into PE pathogenesis increasingly employ multi-omics approaches. These high-throughput technologies enable comprehensive molecular profiling by simultaneously analyzing diverse biomolecules-including genomic, transcriptomic, proteomic, and metabolomic markers-through advanced analytical platforms such as high-performance liquid chromatography–tandem mass spectrometry (HPLC-MS/MS); next-generation sequencing (NGS); nuclear magnetic resonance (NMR) spectroscopy; multiplex immunoassays. Among these approaches, proteomics has emerged as particularly valuable for PE research, providing detailed protein signatures that reveal disease mechanisms and identify clinically relevant biomarkers [15]. The most well-validated biomarker combination is the ratio of soluble fms-like tyrosine kinase-1 (sFlt-1), an anti-angiogenic factor, to Placental growth factor (PlGF), a pro-angiogenic protein. This ratio demonstrates exceptional predictive value, with area-under-the-curve (AUC) values of 0.92 for early-onset PE and 0.87 for late-onset PE.

Clinically, the sFlt-1/PlGF ratio enables PE prediction 4–5 weeks before symptom onset in early cases and 1–2 weeks in late presentations. When combined with standard clinical evaluation, PlGF testing enhances diagnostic accuracy and facilitates earlier intervention [16,17,18]. Compared to ultrasound-based methods, quantitative proteomic analysis of plasma and serum offers distinct advantages, including greater objectivity, superior reproducibility, and reduced operator dependence-characteristics particularly valuable for standardized screening programs.

Recently, Than et al. (2024) proposed a pragmatic molecular theory of PE based on placental and plasma proteomic analyses, identifying four distinct PE subclasses [19]. The placental subclass is characterized by impaired vascular perfusion and a maternal anti-angiogenic state, while the metabolic subclass exhibits hypercoagulability, leading to placental vascular thrombosis. The maternal anti-fetal rejection subclass features CXCL10 overexpression and cytotoxic T-cell infiltration, resulting in inflammatory placental lesions. Finally, the extracellular matrix (ECM)-related subclass, typically associated with late-onset PE, presents with lower mean arterial pressure and higher neonatal birth weight percentiles. This subclass involves dysregulated ECM biochemical interactions, such as altered signaling between collagen receptors and interstitial collagenases (e.g., matrix metalloproteinase-1) [19].

The Fetal Medicine Foundation (FMF) has developed an advanced first-trimester screening algorithm (10–14 weeks’ gestation) that integrates maternal risk factors with biophysical and biochemical markers—including pregnancy-associated plasma protein-A (PAPP-A) and PlGF—to identify high-risk PE pregnancies (>1:100). This model surpasses conventional 11–13 week screening, which detects fewer than 40% of preterm PE cases due to its reliance on biophysical markers alone. Notably, the FMF algorithm demonstrates high sensitivity for the “placental” PE subtype described by Prof. Romero’s group and has been endorsed by the International Federation of Gynecology and Obstetrics (FIGO) as a superior predictive tool [20,21]. Currently, the PlGF-based algorithm is implemented in the Astraia company’s software (Nexus/Astraia, Ismaning, Germany) and has been incorporated into multiple clinical guidelines for routine first-trimester screening.

This systematic review critically evaluates emerging evidence on circulating blood proteins as potential biomarkers for PE, with particular emphasis on the necessity for standardized proteomic analysis protocols in plasma and serum studies. Establishing such methodological consistency could facilitate the development of targeted predictive models and accelerate the translation of proteomic discoveries into clinical applications for PE risk stratification and management.

## 2. Materials and Methods

### 2.1. Literature Search Strategy

This systematic review was performed in accordance with the PRISMA 2020 guidelines and checklist [22]. A systematic literature search was conducted to identify original articles containing data on proteins identified through quantitative proteomic analysis of blood serum or plasma. The search was performed in the PubMed and MEDLINE databases, covering publications from January 2019 to October 2024. The following search string was used: ‘(“proteomics”[Title/Abstract]) AND (“preeclampsia”[Title/Abstract] OR “intrauterine growth restriction”[Title/Abstract] OR “fetal growth restriction”[Title/Abstract])’. Filters were applied to limit results to human studies, original research articles, and publications in English.

### 2.2. Eligibility Criteria

Studies were included if they met all of the following criteria: (1) information was provided on gestational age at the time of blood collection, (2) timing of delivery was reported, and (3) quantitative data were available on protein marker levels comparing PE and/or fetal growth restriction (FGR) and control groups. Studies were also required to utilize quantitative proteomic analysis of maternal blood serum or plasma. If a biomarker was evaluated in multiple cohorts within the same study, each cohort’s results were analyzed separately.

### 2.3. Study Selection Process

Duplicate records were removed using EndNote (version 20.6 Bld 17174, Clarivate, Philadelphia, PA, USA) before screening. In the first screening phase, studies were excluded if they met any of the following criteria: (1) non-original research types, (2) non-pregnant or non-human study populations, (3) fewer than five pregnant women in any study group, or (4) analysis of biological samples other than maternal blood serum or plasma.

**Figure 1:**
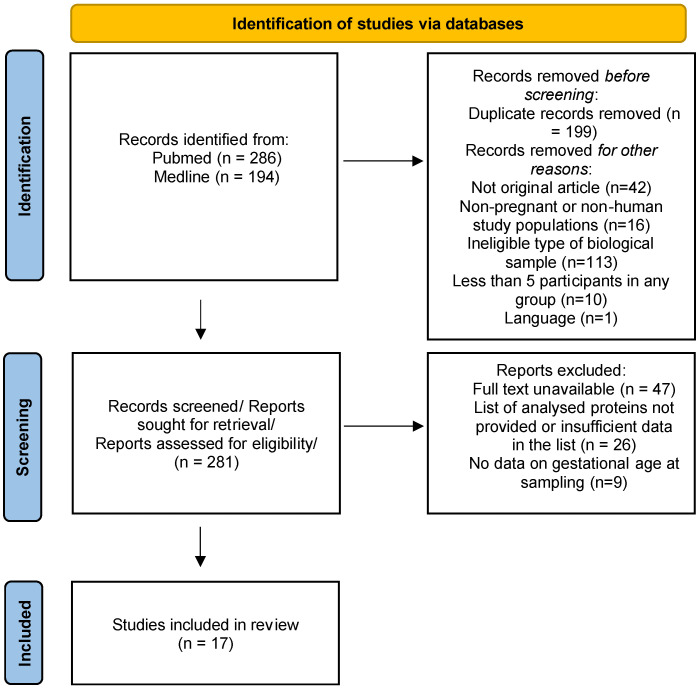
PRISMA 2020 flowchart diagram for systematic review.

In the second selection phase, full-text articles and related supplementary materials flagged as potentially relevant during title/abstract screening were evaluated. Studies were excluded if they met any of the following criteria: (1) did not provide a list of proteins identified through quantitative proteomic analysis of maternal serum in PE and/or FGR compared to healthy controls, (2) lacked data on gestational age at sampling, or (3) had inaccessible full texts (Figure 1).

Two independent reviewers screened all titles and abstracts using predefined criteria. Disagreements were resolved through discussion or, if needed, by a third reviewer. Full texts of potentially eligible studies were independently assessed by the same reviewers.

Ultimately, 17 articles meeting the inclusion criteria were selected for meta-analysis (Table S1). Three comparison groups were established based on PE subtypes: late-onset (healthy women vs. those developing PE after 34 weeks), early-onset (healthy women vs. cases diagnosed before 34 weeks), and mixed-type (healthy women vs. PE cases without onset timing restrictions). Additional comparison groups reflected blood sampling timelines: first trimester (weeks 1–13), second trimester (weeks 14–27), and third trimester (weeks 28–40). The studies included in the meta-analysis may contain multiple independent PE cohorts, representing different PE subtypes. Additionally, some studies collected maternal blood at multiple timepoints during pregnancy. Thus, a single study could contribute multiple comparisons—either due to the inclusion of distinct PE subtypes or repeated blood sampling over time. The extracted variables comprised the following: study identification (lead author names, publication years, and reference details); protein quantification: (fold-change values between PE and control groups with corresponding sample sizes, measurement units, and statistical significance (*p*-values with specified tests)); methodological details: blood matrix (serum/plasma), sampling timing relative to gestation, and concise proteomic workflow description. Clinical parameters (cohort size, maternal age, gestation age at delivery, birth weight) were selected as additional information.

### 2.4. Quality Assessment

The methodological quality of each included study was assessed using the Newcastle–Ottawa Scale (NOS) [23], which evaluates three domains: selection of study groups, comparability between groups, and ascertainment of outcomes. Scores were assigned out of a maximum of 10 points, with higher scores indicating lower risk of bias.

### 2.5. Data Synthesis and Bioinformatics

Proteins identified in at least two independent studies were considered reproducible markers. We assessed two levels of reproducibility: (1) across all studies and (2) within specific trimesters. Reproducible markers were further categorized based on the consistency of their directional changes (matched vs. unmatched). For proteins detected in three or more comparisons, we calculated the range of fold-change alterations.

Reproducible protein markers were functionally characterized using STRING (version 12.0, STRING Consortium) [24] and PANTHER (version 19.0, University of Southern California, Los Angeles, CA, USA) [25] databases. Pathways according STRING database with false discovery rate <0.05 were selected as importance. Data visualization was performed using ggplot2 (3.5.1 version) by R language (version 4.3.1, The R Foundation for Statistical Computing, Viene, Austria) and STRING’s native tools.

For data visualization and synthesis, upper-bound *p*-values were approximated to significance thresholds while preserving precision.

## 3. Results

### 3.1. Quality Assessment, Methodological Trends, and Cohort Characteristics

This meta-analysis, based on a systematic search algorithm, included 17 articles published between January 2009 and October 2024. The included studies were evaluated using a Newcastle–Ottawa Quality Assessment Scale, with a maximum possible score of 10 points. A score of ≥7 was considered indicative of good methodological quality. Based on this criterion, 14 out of 17 studies (82%) were classified as high quality (Table S1). The scores ranged from 6 to 10, with a median value of 8, reflecting an overall acceptable level of methodological rigor across the included literature.

The studies collectively reported 41 comparative analyses of blood proteomes in PE and control groups, with each article containing between one and five comparisons differing by cohort and/or sampling time. Among the included studies, 7 cohorts focused on early-onset PE, 16 on late-onset PE, and 15 did not distinguish PE by time of manifestation, classifying them as mixed cases.

Serum and plasma samples were equally represented across the studies. To enhance the depth of proteome analysis, seven studies employed major protein depletion during sample preparation. Protein fractionation was a critical step, with five studies (29%) utilizing gel-based separation methods such as two-dimensional electrophoresis (2-DE, DIGE) and denaturing electrophoresis (SDS-PAGE). Liquid chromatography coupled with mass spectrometry (LC-MS/MS) emerged as the most widely used technique, appearing in 11 studies (64%) (Table 1).

A notable methodological trend involved structuring studies into multiple phases. The exploratory phase typically relied on semi-quantitative LC-MS/MS to identify potential biomarkers, while the validation phase employed highly sensitive techniques such as LC-MRM-MS with isotope-labeled internal standards and enzyme-linked immunosorbent assay (ELISA) to confirm findings in independent cohorts. Recent advancements have introduced high-throughput proteomic platforms, including those based on chemically modified DNA aptamers (SOMAmer) and highly specific antibodies (Olink), which enable large-scale protein analysis with enhanced specificity and dynamic range. These technologies, which rely on microarray analysis, sequencing, or PCR-based detection, hold significant promise for personalized medicine and pharmacological research.

**Figure 2:**
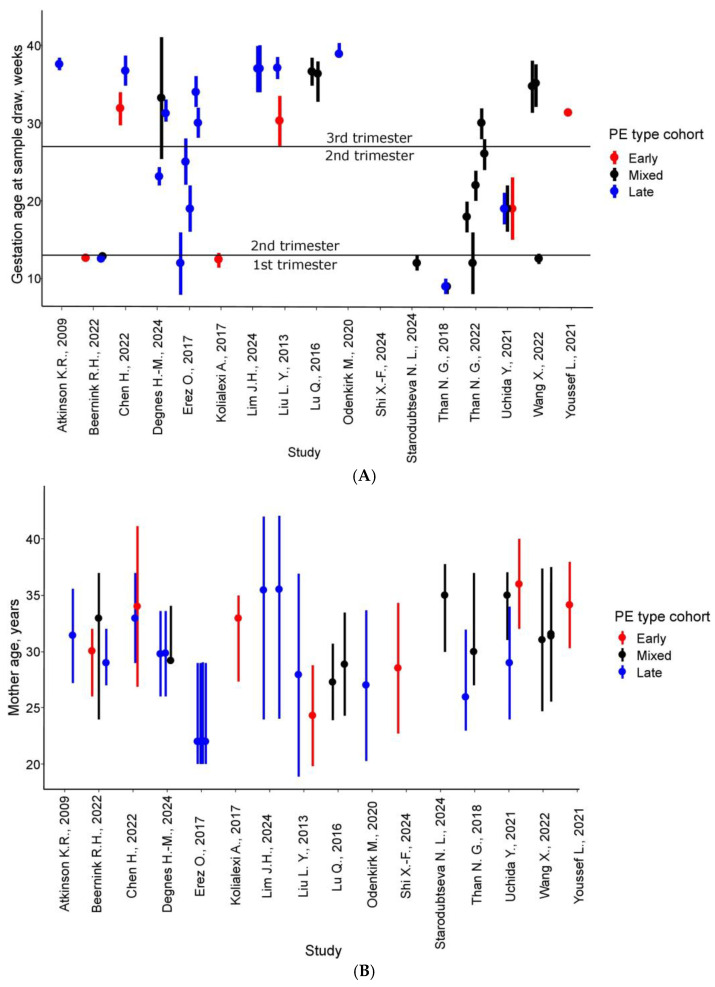

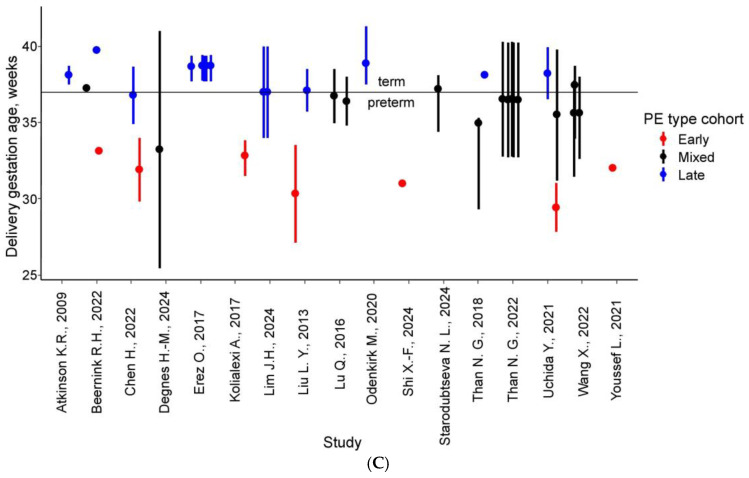
Characteristics of PE study cohorts [26,27,28,29,30,31,32,33,34,35,36,37,38,39,40,41,42]. (**A**) Timing of blood collection for PE protein marker identification. (**B**) Maternal age distribution. (**C**) Gestational age at delivery. Dashed boundaries indicate reported extreme values (range, mean ± SD, or IQR); dots represent weighted central tendency measures (mean, median, or range midpoint).

The median cohort size for PE patients was 35 individuals, with the largest cohorts reported by Than et al., 2022 [39] (n = 109) and Erez et al., 2017 [30] (n = 76) (Table S1, Figure S1). Control groups had a median size of 42 patients, reaching up to 120 in one study. Severe PE was analyzed in 15 comparisons, while FGR, frequently associated with PE, was examined in eight. Sample collection predominantly occurred in the third trimester (>27 weeks) [26,28,29,30,32,33,34,35,41,42], though some studies investigated early PE, with blood draws in the first or second trimester (Figure 2) [27,31,40]. Patient ages ranged from 18.9 to 42 years, with weighted averages falling between 22 and 36 years. In ten comparisons, the average gestational age at delivery exceeded 37 weeks, and eight cohorts reported no preterm births.

### 3.2. PE-Specific Maternal Plasma/Serum Proteomic Profile

**Figure 3:**
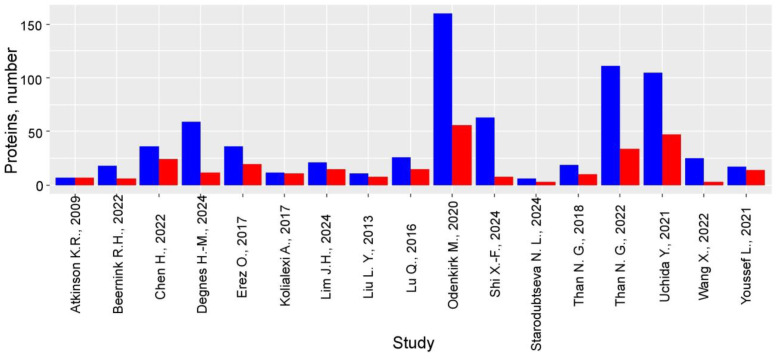
Statistically significant protein alterations (*p* < 0.05) in PE maternal plasma/serum across studies [26,27,28,29,30,31,32,33,34,35,36,37,38,39,40,41,42]. Blue: all markers, identified in each study; red: consistently replicated markers (≥2 independent studied).

The 17 analyzed studies collectively identified 561 proteins with statistically significant alterations in PE, of which 122 were replicated in at least two independent studies (Table S3). Notably, Odenkirk et al. (2020) [35] reported the highest overlap, with 37 consistent protein changes in late third-trimester plasma samples (mean gestational age 38.9 ± 1.4 weeks) (Table S4). A striking confirmation of early findings emerged from Atkinson et al. (2009) [26], where six of seven initially identified proteins were later validated in independent studies (Figure 3, Table S4). The study by Odenkirk et al. (2020) [35] also demonstrated exceptional consistency, with 27 proteins showing uniform directional changes in PE. In contrast, first- and second-trimester studies revealed minimal concordance; for instance, no proteins exhibited consistent changes in the works of Beernink et al. (2022) [27], Degnes et al. (2024) [29], or Uchida et al. (2021) [40]. This striking trimester-specific disparity underscores more pronounced serum/plasma proteome disruptions in late pregnancy, highlighting the need for longitudinal studies to elucidate PE pathogenesis and biomarker potential.

**Figure 4:**
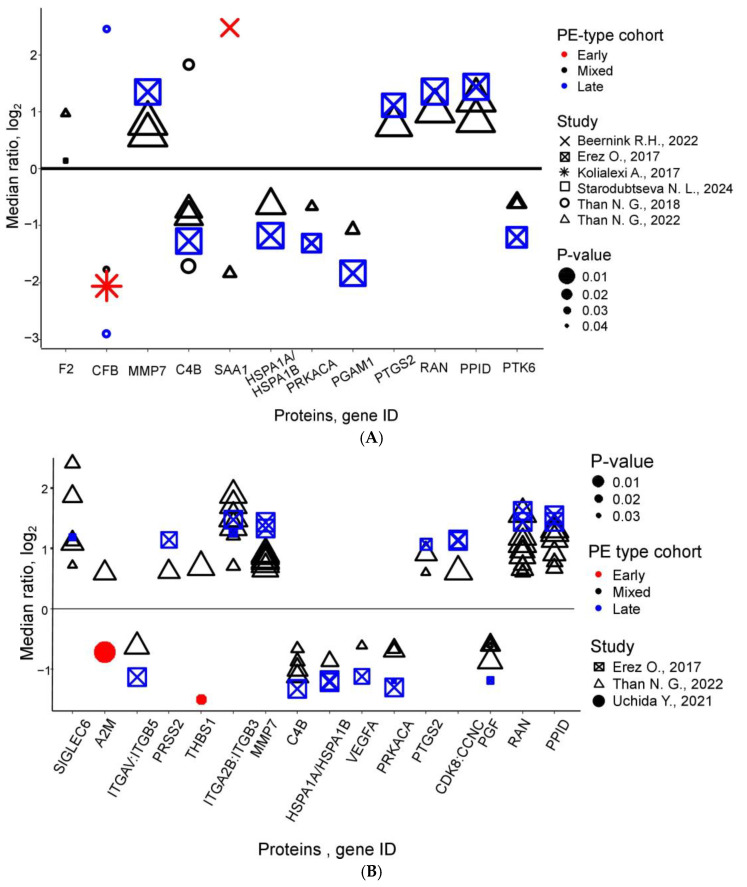

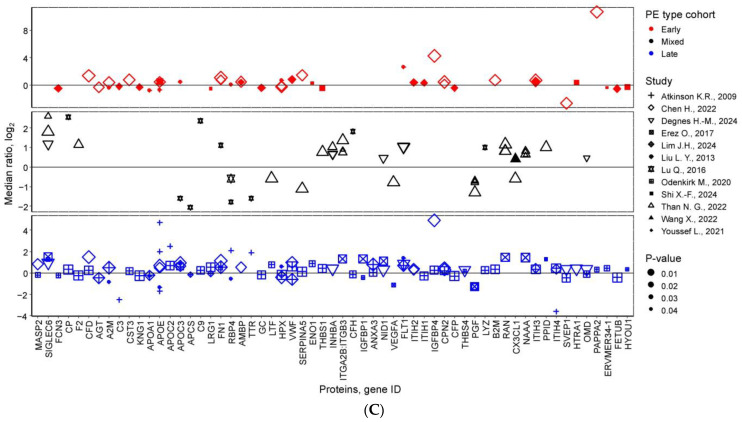
Ratio of protein expression levels (PE/control) for replicated PE markers (identified in ≥2 studies) plotted on a logarithmic scale: (**A**) first trimester [27,30,31,37,38,39], (**B**) second trimester [30,39,40], (**C**) third trimester [26,28,29,30,32,33,34,35,36,39,41,42]. Color coding distinguishes PE subtypes: red for early PE, blue for late PE, and black for mixed PE. Point shapes represent the originating studies, while point size corresponds to statistical significance—larger points indicate stronger significance (lower *p*-values in the original study).

Early-pregnancy proteomics yielded 12 recurrently altered proteins (Figure 4A). Several proteins—including Prothrombin, Matrilysin, Prostaglandin G/H synthase 2, and Peptidyl-prolyl cis-trans isomerase D—demonstrated consistent increases, while others like Heat shock 70 kDa protein 1A/1B, cAMP-dependent protein kinase catalytic subunit alpha, Phosphoglycerate mutase 1 and Protein-tyrosine kinase 6 decreased uniformly. Intriguingly, proteins such as Complement factor B and Serum amyloid A-1 exhibited bidirectional changes, reflecting PE’s pathological complexity (Table 2, Tables S5 and S6).

Second-trimester meta-analysis identified 16 candidate biomarkers according to at least two studies (Figure 4B), with proteins like Sialic acid-binding Ig-like lectin 6, Trypsin-2, Integrin alpha-IIb: beta-3 complex, Matrilysin, Prostaglandin G/H synthase 2, Cyclin-dependent kinase 8:Cyclin-C complex, GTP-binding nuclear protein Ran, and Peptidyl-prolyl cis-trans isomerase D consistently elevated. Integrin alpha-V: beta-5 complex, Complement C4b, Heat shock 70 kDa protein 1A/1B, Vascular endothelial growth factor A, isoform 121, cAMP-dependent protein kinase catalytic subunit alpha, and Peptidyl-prolyl cis-trans isomerase D showed decreased levels (Table S7). However, conflicting data for proteins like Alpha-2-macroglobulin and Thrombospondin-1—observed by Uchida et al. (2021) [40] and Than et al. (2022) [39]—highlight methodological and cohort variability challenges (Table S8).

Third-trimester studies revealed 59 recurrently altered proteins (Figure 4C), with early PE showing consistent increases in maternal blood Fibronectin, Protein AMBP, Inter-alpha-trypsin inhibitor heavy chain H3, and Carboxypeptidase N subunit 2. Late PE exhibited more complex patterns: 15 proteins (e.g., Fibronectin, Vascular endothelial growth factor receptor 1, Inter-alpha-trypsin inhibitor heavy chain H3 and H2) were elevated, 2 (Apolipoprotein A-I, PlGF) decreased, and 11 (including Apolipoprotein E and Inter-alpha-trypsin inhibitor heavy chain H4) showed discordant results across studies (Tables S9 and S10). These trimester-specific signatures and inconsistencies underscore the need for standardized methodologies and refined clinical stratification in PE proteomics research.

### 3.3. Bioinformatics Analysis

Several proteins identified as PE markers in at least two first-trimester studies—prothrombin, serum amyloid A-1 protein (SAA1), complement C4b, and complement factor B—demonstrate statistically significant associations with kidney disease, while prothrombin and C4b are additionally linked to protein S deficiency (Table S11). A key pathological feature of early PE development is complement system activation (Figure S2). Furthermore, dysregulation of metabolic, hormonal, and neuronal signaling pathways—including those mediated by cAMP-dependent protein kinase A (PRKACA)—plays a critical role in PE pathogenesis (Figure S3).

In the second trimester of pregnancy, PE progression involves pronounced immune dysregulation, particularly complement activation, alongside disruptions in angiogenesis and ECM remodeling. These processes are mediated by integrin-dependent signaling and PI3K/AKT/mTOR cascade activation (Figure S4). Molecular dysregulation patterns resemble those of the first trimester but intensify (Figure S3). Notably, several implicated proteins contribute to fetal developmental processes, including morphogenesis, tube formation, and primary germ layer establishment, with nine proteins expressed in embryonic structures (Table S12). Pathway analyses reveal significant enrichment in cardiomyopathy-related pathways (Figure S3), suggesting a potential link between PE and cardiovascular disorders.

The maternal protein profile exhibits the most pronounced alterations as PE approaches clinical onset. PE-associated markers in this stage correlate not only with eclampsia but also with kidney disease and coagulation disorders (Table S13). Key pathways include complement/coagulation cascades and insulin-like growth factor (IGF) transport regulation by insulin-like growth factor-binding proteins (IGFBPs) (Figure S5, Table S13). Strikingly, 17 PE serum markers are expressed in placental tissues (Table S13). PANTHER analysis highlights coagulation imbalances, metabolic disruptions, and inflammatory signaling dysregulation as dominant features of PE in the third trimester of pregnancy (Figure S3).

## 4. Discussion

The meta-analysis incorporated studies published between 2009 and 2024 that reported quantitative proteomic analyses of serum and plasma from pregnant women with PE (n = 842) compared to controls with uncomplicated pregnancies (n = 1148). This review systematically evaluated key parameters including patients’ clinical and demographic characteristics, sample collection timing, and PE subtypes (early onset, late onset, and mixed forms). Notably, serum Complement C4b levels demonstrated a consistent reduction across mixed and late-onset PE cohorts during early gestation, suggesting it as a promising candidate for the early detection of preeclampsia. Across the 17 selected studies, researchers identified 122 proteins showing statistically significant expression level alterations in PE cases versus controls, with each protein confirmed by at least 2 independent studies.

### 4.1. First-Trimester Predictive Biomarkers of PE

Complement system hyperactivation emerges as one of the most significantly enriched biological processes in first-trimester PE, playing a crucial role in fetoplacental immunological tolerance maintenance [43]. Disruption of complement homeostasis—whether through excessive activation or pathway dysregulation—represents a key mechanism in pregnancy complications [44]. This aberrant activation triggers a cascade of pathological events: systemic inflammation, vascular endothelial damage, and subsequent endothelial dysfunction manifesting as hypertension and proteinuria. Furthermore, it elevates oxidative stress and disrupts placental immune regulation, ultimately impairing trophoblast invasion and placental development [43,45,46,47].

The complement system serves as a critical interface between innate immunity and physiological homeostasis, participating in tissue development/repair and interacting with multiple endogenous systems (renin-angiotensin, coagulation, and kallikrein-kinin pathways). Comprising over 50 membrane-bound and circulating proteins, its dysregulation shows distinct biomarker patterns in PE. Notably, serum Complement C4b levels demonstrate consistent reduction across mixed and late-onset PE cohorts during early gestation [30,38,48]. Balduit et al. (2024) attribute this finding to enhanced classical/lectin pathway activity, where increased C4 proteolysis elevates C4b cleavage products [49]. Similarly, Complement Factor B (alternative pathway) shows marked depletion in early pregnancy, independent of PE onset timing [31,38]. These observations support growing interest in complement-targeted interventions for PE prevention and treatment [50,51], with low-dose aspirin-the only internationally recommended PE prophylactic-demonstrating specific inhibitory effects on alternative pathway activity [52,53].

Complement activation is closely linked to coagulation cascades. This complement-coagulation axis manifests in proteomic signatures: meta-analyses reveal elevated first-trimester Prothrombin levels in PE cases versus controls [37,48], while Nguyen et al. (2019) report reduced Fibrinogen activity, suggesting a thrombogenic shift toward fibrin formation [54]. Such findings underscore the bidirectional relationship between complement activation and hemostasis in endothelial dysfunction [55,56].

The first-trimester proinflammatory and hypercoagulable state may induce multiorgan dysfunction, particularly affecting hepatic and renal systems. Associated protein markers correlate with renal impairment and Protein S deficiency (Table S11). Renal dysfunction exacerbates PE risk through multiple mechanisms: impaired pressure natriuresis, fluid retention, and uremic toxin accumulation collectively increase vascular stress. Concurrent Protein S deficiency-by reducing natural anticoagulant activity-potentiates hypercoagulability, placental microthrombosis, and ischemia-driven antiangiogenic factor release, creating a vicious cycle of placental dysfunction.

In conclusion, Prothrombin, Matrilysin (MMP7), Prostaglandin G/H synthase 2 (COX-2), and Peptidyl-prolyl cis-trans isomerase D (PPID) showed consistent increases across studies, while Heat shock 70 kDa protein 1A/1B (HSPA1A/B), cAMP-dependent protein kinase catalytic subunit alpha (PRKACA), Phosphoglycerate mutase 1 (PGAM1), and Protein-tyrosine kinase 6 (PTK6) exhibited uniform decreases. Considering that early-onset PE is generally associated with a more severe clinical course compared to late-onset PE, and that the majority of the identified biomarkers were detected in studies focusing on early or mixed PE cohorts, it can be inferred that alterations in concentrations of these proteins are significantly associated with more severe forms of the disease. Together, these findings highlight a panel of promising biomarkers rather than a single candidate, reflecting the multifactorial nature of PE.

### 4.2. Blood Proteome Alterations in Second-Trimester PE

Emerging proteomic evidence reveals distinct patterns of integrin dysregulation in PE, with the Integrin αIIbβ3 complex (GPIIb/IIIa) showing consistent elevation during late gestation across multiple studies, while Integrin αVβ5 demonstrates reciprocal downregulation. These transmembrane receptors, critical for cellular adhesion and signaling, play pivotal roles in placental physiology. The platelet-specific GPIIb/IIIa complex becomes activated by thrombin and collagen, enhancing its binding affinity for fibrinogen and von Willebrand factor to promote platelet aggregation and microthrombosis-a prothrombotic state that exacerbates placental hypoperfusion and ischemic injury, central features of PE pathophysiology [57]. While low-dose aspirin prophylaxis effectively reduces platelet aggregation in high-risk pregnancies, the potential therapeutic use of targeted GPIIb/IIIa inhibitors like abciximab requires further investigation for pregnancy-specific safety and efficacy [13].

Concurrent with integrin dysregulation, matrix metalloproteinase-7 (MMP-7) is significantly elevated in both first- and second-trimester mixed and late-onset PE cases [58,59]. Its critical involvement in ECM remodeling during placentation positions MMP-7 as a promising biomarker for PE [30,58,59]. Similarly, the sialic acid-binding lectin Siglec-6 is markedly overexpressed in PE syncytiotrophoblasts [60,61]. However, its detection in maternal circulation relies on advanced SOMAmer-based proteomic techniques rather than conventional assays [30,39]. Siglec-6 interactions with leptin and PlGF implicates it in fetomaternal immune dysregulation [60,61], though clinical utility hinges on the development of ultrasensitive detection methods and large-scale validation studies.

Vascular endothelial growth factor A (VEGF-A), PlGF, and sFlt-1 are among the most extensively studied biomarkers in PE. Our meta-analysis reveals that PlGF levels are significantly reduced in the second and third trimesters of pregnancies complicated by PE. Similarly, VEGF-A levels decline—during the second trimester in late-onset PE and the third trimester in both early- and late-onset PE. These alterations reflect an imbalance in vascular growth regulation, contributing to placental dysfunction and the hallmark symptoms of PE, including hypertension and proteinuria. Moreover, the elevated sFlt-1 levels observed in PE exacerbate this imbalance by neutralizing the biological activity of VEGF-A and PlGF, supporting the hypothesis that PE arises from endothelial dysfunction driven by impaired angiogenic signaling [62,63].

This vascular pathology aligns with broader cardiovascular manifestations revealed through KEGG pathway analysis, which shows significant enrichment of cardiomyopathy-related pathways in PE [64,65]. The condition’s cardiovascular impact becomes particularly evident by mid-gestation, when failed hemodynamic adaptation manifests as pathological cardiac remodeling including left ventricular concentric hypertrophy and diastolic dysfunction, accompanied by elevated biomarkers of myocardial strain such as NT-proBNP and troponin, even in the absence of overt heart failure [66,67]. Most concerning are the long-term implications: women with PE face a four-fold increased risk of subsequent heart failure and double the mortality from ischemic heart disease and stroke [65], underscoring the persistent cardiovascular consequences of this pregnancy complication and the critical need for ongoing postpartum monitoring and targeted prevention strategies.

### 4.3. Third-Trimester Protein Biomarkers of PE

The data obtained demonstrate substantially more dysregulated serum/plasma proteins in third-trimester (59 proteins) compared to the second (16 proteins) and first trimesters (12 proteins), with each protein alteration confirmed by at least two independent studies. This temporal pattern reflects both biological and methodological factors. From a practical standpoint, third-trimester biomarker research benefits from easier cross-sectional sampling compared to the logistical challenges of longitudinal studies requiring early biospecimen collection for a condition with only ~5% incidence. Biologically, the third trimester represents the culmination of progressive pathological processes-including placental dysfunction, endothelial impairment, and systemic inflammation-which reach maximal intensity as clinical symptoms emerge.

The proteomic heterogeneity observed in late gestation enables identification of distinct biomarker patterns corresponding to different PE subtypes, providing critical insights into disease mechanisms and potential therapeutic targets. Notably, many third-trimester protein alterations are linked not only to PE but also to renal and coagulation disorders, with evidence suggesting these pathophysiological changes originate as early as the first trimester. Complement and coagulation cascade dysregulation, central to PE pathogenesis, demonstrates measurable changes from pregnancy onset. Additionally, third-trimester biomarkers reveal significant enrichment in IGF transport and signaling pathways, underscoring the importance of metabolic dysregulation in PE development.

Metabolic disturbances in PE encompass impaired lipid and carbohydrate metabolism, insulin resistance, and IGF dysregulation, all contributing to endothelial dysfunction, systemic inflammation, and placental insufficiency. Even without overt diabetes, PE patients frequently exhibit insulin resistance leading to abnormal glucose/lipid metabolism that exacerbates oxidative stress and vascular impairment [68,69,70,71,72,73,74]. PE specific metabolic changes include elevated free fatty acids and triglycerides correlating with disease severity [75], along with lipid deposition in spiral arteries resembling early atherosclerotic changes [76].

Our systematic review and meta-analysis revealed multiple maternal blood proteins with consistent and significant alterations during early pregnancy in women who later developed PE. Strikingly, several of these proteins—including GTP-binding nuclear protein Ran, peptidyl-prolyl cis-trans isomerase D (PPID), protein-tyrosine kinase 6 (PTK6), and phosphoglycerate mutase 1 (PGAM1)—have rarely or never been previously associated with PE. Notably, these dysregulations were detectable as early as the first trimester, even in cohorts that subsequently developed late-onset or mixed-type PE. Ran and PPID levels were consistently elevated, whereas PTK6 and PGAM1 were reduced, suggesting their potential as novel early biomarkers or mechanistic contributors to PE pathogenesis. Further studies are needed to validate their clinical utility and elucidate their pathophysiological roles.

Nine proteins involved in IGF transport and signaling show consistent directional changes in third-trimester PE across multiple studies: Apolipoprotein A-I, Ceruloplasmin, Insulin-like growth factor-binding protein 4, Fibronectin, Inter-alpha-trypsin inhibitor heavy chain H2, Pappalysin-2, and Cystatin-C are elevated, while Complement C3 and Kininogen-1 are decreased. Apolipoprotein A-I, the major HDL component crucial for cholesterol transport and vascular protection, becomes dysfunctional in PE despite increased antioxidant capacity-a likely compensatory response to oxidative stress [77,78,79,80]. Ceruloplasmin elevation reflects both oxidative stress and inflammation, with levels correlating with PE severity as this ferroxidase attempts to mitigate free iron-mediated ROS generation [81,82,83,84,85].

Fibronectin, a multifunctional ECM protein critical for placentation, exhibits profound alterations in PE [86,87,88,89]. Although plasma fibronectin levels typically rise modestly during normal pregnancy (31.8% increase), PE patients display markedly elevated concentrations (94.5% increase) [90,91]. Glycosylated fibronectin stands out as a particularly promising early biomarker, detectable in first-trimester samples from women who subsequently develop PE and strongly associated with adverse outcomes, including hypertension, preterm delivery, and FGR [92]. Notably, when integrated into first-trimester screening algorithms, glycosylated fibronectin significantly enhances preterm PE detection rates, improving sensitivity from 64.9% to 82.9% in an Asian cohort [93].

### 4.4. Standardization of Blood Protein Analysis: Current Challenges and Future Directions for PE Diagnosis and Prediction

Accurate analysis of blood proteins is critical for PE prediction and diagnosis, yet significant concerns persist regarding measurement reliability. A major challenge in plasma/serum proteomics is the substantial inter-laboratory variability in measured protein concentrations (or concentration ratios). These inconsistencies arise from multiple sources: lack of standardized pre-processing protocols (sample collection, handling, and storage); heterogeneity in sample preparation methods (e.g., protein depletion strategies, labeling techniques); diverse fractionation approaches (SDS-PAGE, 2D electrophoresis, preparative LC); variability in detection platforms (HPLC-MS/MS, HPLC-MRM-MS, ELISA, SOMAmer, Olink) [94]. Furthermore, the high correlation between results from Erez O et al. [30] and Than N et al. [48]—both conducted at the same research center—along with their limited overlap of common proteins with other studies, highlights the significant impact of inter-laboratory variation.

The choice of sample type represents a critical methodological consideration, as plasma and serum yield divergent protein profiles due to inherent processing differences. Plasma is the preferred matrix for analyzing complement system proteins, as it circumvents artifactual in vitro activation during serum coagulation and fibrinolysis [95]. Sample preparation must also account for protein–protein interactions, particularly those involving high-abundance species like albumin and apolipoproteins, which can mask or alter biomarker detection [96,97,98]. Furthermore, pre-analytical variables—including storage conditions, thawing protocols, and freeze–thaw cycles—introduce additional variability that must be rigorously controlled to ensure data reproducibility [99,100].

Inconsistent sampling timelines across studies further complicate standardization and hinder meta-analyses. Most studies in our systematic review and meta-analysis measured protein levels at PE diagnosis (third trimester), explaining the scarcity of validated first/second-trimester predictive markers. Notably, five first/second-trimester studies [27,31,37,38,40] were limited by extremely small PE cohort sizes, significantly reducing the statistical power and reliability of their comparative analyses. These findings highlight the urgent need for multicenter longitudinal studies with early-pregnancy sampling (8–12 weeks) and integration of molecular subtyping to account for PE heterogeneity. Given PE’s syndromic nature, no single sampling strategy can predict all pathological variants [101]. Additional confounding factors include cohort heterogeneity (ethnic composition, sample size), disease severity (mild/severe), onset timing (early/late PE), and comorbid conditions.

The identified biomarkers hold significant potential to enhance clinical screening for PE. They could be integrated into first-trimester multi-marker panels alongside established predictors—such as maternal risk factors, mean arterial pressure, uterine artery Doppler indices, and biochemical markers (PAPP-A, PlGF, and β-hCG)—to improve early risk assessment. Beyond screening, these biomarkers may enable more precise individualized risk stratification, particularly for women classified as intermediate risk after standard evaluation. They could also improve early detection of late-onset PE, where current methods lack sensitivity. Additionally, serial monitoring of biomarker dynamics during early pregnancy could help assess biological responses to preventive therapies (e.g., low-dose aspirin), allowing timely adjustments in clinical management. From a research perspective, these biomarkers—linked to cellular stress, protein folding, intracellular transport, and complement regulation—provide novel insights into PE pathophysiology and highlight potential therapeutic targets. Simplified biomarker panels incorporating these proteins could further facilitate the development of point-of-care diagnostic tools, improving accessibility across diverse healthcare settings. However, prospective validation studies remain essential to confirm their clinical utility.

The identified biomarkers could significantly enhance personalized PE management through multiple avenues. By detecting molecular alterations early, these biomarkers may enable precise risk stratification and facilitate tailored preventive strategies—such as low-dose aspirin therapy or intensified monitoring—based on individual patient profiles. Serial assessment of biomarker levels could further provide real-time insights into treatment response, allowing clinicians to adapt management approaches dynamically throughout pregnancy. Beyond clinical applications, these biomarkers’ roles in critical biological pathways—including complement activation, cellular stress response, and protein folding—offer promising opportunities to identify novel therapeutic targets and develop targeted, mechanism-driven interventions.

Recent genomic and proteomic research has identified four molecular PE subtypes: canonical/placental (primarily angiogenic dysfunction); metabolic (associated with obesity/insulin resistance); immunological (maternal/fetal immune dysregulation features); maternal (preexisting cardiovascular/metabolic risk) [38,39,48,102]. These subtypes align with distinct maternal hemodynamic patterns observed across hypertensive pregnancy disorders [103]. Development of subtype-specific PE diagnostic models improved quality of PE prevention. Advancing PE diagnostics requires standardized analytical protocols for sample collection/processing, large multicenter studies incorporating molecular subtyping, and longitudinal designs tracking clinical progression.

To advance methodological standardization in preeclampsia proteomics research, several strategic approaches could be implemented. An international collaborative consortium could be established to unify protocols for sample collection, processing, and data analysis across research institutions. Drawing inspiration from established standards such as MIAME and PRIDE, the field would benefit from developing specialized reporting guidelines to enhance transparency and reproducibility. The creation of shared reference datasets from rigorously characterized PE cohorts would provide valuable validation benchmarks, complemented by interlaboratory proficiency testing to systematically assess and reduce technical variability. Furthermore, aligning these standardization efforts with existing clinical networks like ISSHP or PREPARE would facilitate smoother translation of research findings into clinical practice.

Importantly, this study demonstrates several methodological strengths that reinforce the reliability of its findings. The systematic review was conducted in accordance with the PRISMA 2020 guidelines, and the quality of included studies was systematically evaluated using a modified Newcastle–Ottawa Scale. The majority of studies (82%) achieved a score of 7 or higher out of 10, indicating generally high methodological rigor. Clinical and proteomic data were sufficiently detailed to permit stratified analysis by gestational age and PE subtype. Moreover, the incorporation of studies utilizing advanced high-throughput proteomic platforms, coupled with orthogonal validation approaches, enhances the translational value of the identified protein markers. Nonetheless, limitations remain, including substantial heterogeneity in sample processing protocols and proteomic methodologies, which prevented effect-size pooling in a formal meta-analysis. Early-pregnancy datasets were limited and displayed poor overlap, constraining conclusions regarding first- and second-trimester biomarkers. These findings emphasize the ongoing need for standardized, multi-phase study designs with longitudinal sampling and unified analytical pipelines across laboratories.

## 5. Conclusions

As a cornerstone discipline of modern biomedical research, proteomics has achieved unprecedented analytical power through the integration of cutting-edge technologies. Contemporary platforms—including high-resolution LC-MS, modified DNA aptamers (SOMAmer), and multiplexed antibody arrays (Olink)—now enable both comprehensive identification and precise quantification of thousands of proteins in complex biological matrices like blood serum and plasma. These technological breakthroughs, coupled with automated sample handling and standardized processing protocols, have transformed proteomics into a robust tool for generating clinically actionable data.

Our comprehensive meta-analysis demonstrates that blood proteome alterations represent a powerful resource for PE prediction, early detection, and mechanistic understanding. These molecular fingerprints not only enhance our understanding of disease pathogenesis but also create new opportunities for developing personalized therapeutic and preventive strategies. These findings emphasize the importance of precision medicine in pregnancy management, grounded in robust molecular data and deep understanding of disease mechanisms.

The refined list of potential PE biomarkers presented in this study provides a valuable foundation for translational research. Key validation strategies include orthogonal verification using Western blotting or ELISA, high-throughput validation via targeted proteomics (SRM/MRM), and multicenter replication studies to assess generalizability. Targeted proteomic approaches offer particular advantages, enabling simultaneous quantification of multiple candidate markers with high specificity and throughput—a crucial step toward clinical implementation.

The clinical utility of proteomic findings requires rigorous standardization across all analytical phases–from pre-analytical processing to data integrity. Only through strict adherence to these principles can we ensure the reproducibility and clinical relevance of proteomic data. The integration of automated systems and artificial intelligence-assisted analysis will further enhance reliability as the field progresses toward routine clinical application.

## Figures and Tables

**Table 1:** Clinical and proteomic data from 17 studies included in the meta-analysis.

Article	Sample Collection, Weeks	PE (n)	Control (n)	Delivery, Weeks **	Sample Type	Proteins *	Matched Proteins **	Method
Atkinson, K.R., 2009 [26]	37.6 ± 0.8	24	24	38.1 ± 0.6	plasma, serum	7	6	Depletion of 6 major plasma proteins, 2-DE, 2D-DIGE, LC-MS/MS
Beernink, R.H., 2022 [27]	12.7	9	9	33.1	serum	6	2	SDS-PAGE, LC-MS/MS
	12.8	8	8	37.2	serum	4	2	
	12.5	6	6	39.7	serum	8	2	
Chen, H., 2022 [28]	31.9 ± 2.1	17	18	31.9 ± 2.1	plasma	26	18	LC-MS/MS
	36.8 ± 1.9	11	18	36.8 ± 1.9	plasma	20	14	
	31.9 ± 2.1	17	18	31.9 ± 2.1	plasma	9	7	
	36.8 ± 1.9	11	18	36.8 ± 1.9	plasma	13	10	
Degnes, H.-M., 2024 [29]	23.2 ± 1.2	35	70	≥34	plasma	2	0	SOMAmer-4979 proteins
	31.2 ± 1.0	35	70	≥34	plasma	60	13	
	25.4–41	37	75	25.4–41	plasma	35	10	
Erez, O., 2017 [30]	28.1–32	76	90	38.7 (37.7–39.4)	plasma	15	9	SOMAmer-1125 proteins
	8.0–16.0	76	90	38.7 (37.7–39.4)	plasma	24	16	
	16.1–22	76	90	38.7 (37.7–39.4)	plasma	26	17	
	22.1–28	76	90	38.7 (37.7–39.4)	plasma	18	12	
	32.1–36	76	90	38.7 (37.7–39.4)	plasma	15	8	
Kolialexi, A., 2017 [31]	12.5 (11.4–13.3)	10	40	32.2 (28.8–37.3)	plasma	11	11	2-DE, MALDI-MS/MS
	12.5 (11.4–13.3)	10	40	32.2 (28.8–37.3)	plasma	4	2	ELISA
Lim, J.H., 2024 [32]	37 (34–40)	26	29	37 (34–40)	plasma	21	15	Depletion of 14 major plasma proteins, LC-MRM-MS with IS (41 proteins)
	37 (35–40)	10	30	37 (35–40)	plasma	3	2	ELISA (3 proteins)
Liu, L. Y., 2013 [33]	30.3 ± 3.2	15	16	30.3 ± 3.2	serum	11	8	ELISA (22 proteins)
	37.1 ± 1.4	17	16	37.1 ± 1.4	serum	11	8	
Lu, Q., 2016 [34]	36.4 ± 1.6	10	10	36.4 ± 1.6	serum	26	15	Depletion of major plasma proteins, SDS-PAGE, LC-MS/MS
	36.7 ± 1.8	69	60	36.7 ± 1.8	serum	1	1	ELISA (1 protein)
Odenkirk, M., 2020 [35]	38.9 ± 1.4	48	98	38.9 ± 1.4	plasma	160	56	Depletion of 14 major plasma proteins, LC-IMS-MS
Shi, X.-F., 2024 [36]	31	42	58	31	serum	63	8	Depletion of major plasma proteins, iTRAQ, high pH RPLC, LC-MS/MS
Starodubtseva, N.L., 2024 [37]	11.0–13.0	30	13	37.2 [34.4; 38.1]	serum	6	3	LC-MRM-MS/MS with IS (125 proteins)
Than, N.G., 2018 [38]	9 (8–9)	5	5	34.9 (29.3–35.3)	serum	14	8	Depletion of 14 major plasma proteins, 2D-DIGE, LC-MS/MS
	9 (8–10)	5	5	38.1 (38.0–38.1)	serum	8	5	
Than, N.G., 2022 [39]	8–15.9	109	90	39.6 ± 1.17	plasma	28	14	SOMAmer-1125 proteins
	16–19.9	109	90	39.6 ± 1.17	plasma	60	19	
	20–23.9	109	90	39.6 ± 1.17	plasma	43	17	
	24–27.9	109	90	39.6 ± 1.17	plasma	19	8	
	28–31.9	109	90	39.6 ± 1.17	plasma	38	14	
Uchida, Y., 2021 [40]	19 ± 4	7	14	29.4 ± 1.6	plasma	105	47	LC-SWATH-MS/MS
	19 ± 3	36	120	35.5 ± 4.3	plasma	4	2	LC-SRM-MS/MS with IS (4 proteins)
	19 ± 2	36	54	38.2 ± 1.7	plasma	2	1	LC-SRM-MS/MS with IS (2 proteins)
Wang, X., 2022 [41]	34.7 ± 3.3	15	15	35.6 [31.4; 37.7]	plasma	25	3	Olink Inflammation panel (92 proteins)
	35.1 [32.1; 37.6]	43	44	35.6 [32.6; 38.0]	plasma	1	0	ELISA (1 protein)
	12.5 ± 0.7	37	37	37.4 [33.9; 38.7]	plasma	1	0	ELISA (1 protein)
Youssef, L., 2021 [42]	31.3	14	6	32	serum	17	14	Depletion of 7 major plasma proteins, TMT, LC-MS/MS

*—Number of significantly altered proteins (*p* < 0.05); **—number of significantly altered proteins replicated in ≥2 studies (*p* < 0.05). LC-MS/MS—liquid chromatography–tandem mass spectrometry, SWATHs—sequential window acquisition of all theoretical mass spectra, SRM—selected reaction monitoring, MRM—multiple reaction monitoring, TMT—tandem mass tag, iTRAQ—isobaric tags for relative and absolute quantitation, IS—isotope-labeled internal standards (tryptic peptides), 2D-DIGE—two-dimensional difference gel electrophoresis with fluorescent labeling, 2-DE—two-dimensional gel electrophoresis, SDS-PAGE—polyacrylamide gel electrophoresis with sodium dodecyl sulfate, SOMAmer—slow off-rate modified DNA aptamers, Olink Inflammation panel—a panel of highly specific antibodies for the analysis of inflammatory markers in plasma/serum, ELISA—enzyme-linked immunosorbent assay. Data presentation: X–Y: range (minimum–maximum); X (Y–Z): mean (minimum–maximum); X ± Y: mean ± standard deviation; X [Y; Z]: median [1st quartile; 3rd quartile].

**Table 2:** Consistently altered proteins in maternal plasma/serum across PE cohorts (≥3). PE subtypes: p—early, m—mixed, l—late onset.

Protein Name	Uniprot ID	Gene ID	Trimester (PE Type)	Number of Comparisons	Fold Change (Min–Max)	References
Complement C4b	P0C0L5	C4B	1 (m, l)	4	0.30–0.61	[30,38,39]
			2 (m, l)	5	0.39–0.63	[30,39]
Complement factor B	P00751	CFB	1 (e, m, l)	3	0.13–0.29	[31,38]
Matrilysin	P09237	MMP7	1 (m, l)	3	1.50–2.55	[30,39]
			2 (m, l)	9	1.58–2.70	[30,39]
Peptidyl-prolyl cis-trans isomerase D	Q08752	PPID	1 (m, l)	3	1.78–2.71	[30,39]
			2 (m, l)	8	1.60–2.93	[30,39]
Protein-tyrosine kinase 6	Q13882	PTK6	1 (m, l)	3	0.43–0.66	[30,39]
GTP-binding nuclear protein Ran	P62826	RAN	2 (m, l)	11	1.52–3.07	[30,39]
			3 (m, l)	4	1.75–2.74	[30,39]
Integrin alpha-IIb: beta-3 complex	P08514:P05106	ITGA2B:ITGB3	2 (m, l)	8	1.64–3.68	[30,39]
			3 (m, l)	4	1.66–2.56	[30,39]
Sialic acid-binding Ig-like lectin 6	O43699	SIGLEC6	2 (m, l)	6	1.65–5.33	[30,39]
			3 (m, l)	6	1.94–6.04	[29,30,39]
Placenta growth factor	P49763	PGF	2 (m, l)	6	0.44–0.66	[30,39]
			3 (m, l)	6	0.41–0.63	[30,39]
cAMP-dependent protein kinase catalytic subunit alpha	P17612	PRKACA	2 (m, l)	4	0.41–0.63	[30,39]
Heat shock 70 kDa protein 1A/1B	P0DMV8/P0DMV9	HSPA1A/HSPA1B	2 (m, l)	3	0.43–0.55	[30,39]
Prostaglandin G/H synthase 2	P35354	PTGS2	2 (m, l)	3	1.52–2.10	[30,39]
Cyclin-dependent kinase 8:Cyclin-C complex	P49336:P24863	CDK8:CCNC	2 (m, l)	3	1.53–2.19	[30,39]
Inter-alpha-trypsin inhibitor heavy chain H2	P19823	ITIH2	3 (e, l)	3	1.22–1.31	[28,42]
Inter-alpha-trypsin inhibitor heavy chain H3	Q06033	ITIH3	3 (e, l)	6	1.22–1.60	[28,35,42]
Vascular endothelial growth factor receptor 1	P17948	FLT1	3 (e, m, l)	6	1.60–6.15	[29,32,33]
Apolipoprotein A-I	P02647	APOA1	3 (e, l)	3	0.56–0.86	[32,33]
Apolipoprotein C-III	P02656	APOC3	3 (e, l)	5	1.33–1.92	[28,33,35]
Apolipoprotein E	P02649	APOE	3 (e, l)	6	1.36–26.0	[26,28,36]
Carboxypeptidase N subunit 2	P22792	CPN2	3 (e, l)	5	1.13–1.39	[28,35]
Hemopexin	P02790	HPX	3 (e, l)	5	0.76–0.91	[28,35]
Inhibin beta A chain	P08476	INHBA	3 (m, l)	5	1.31–2.04	[29,39]
N-acylethanolamine-hydrolyzing acid amidase	Q02083	NAAA	3 (m, l)	5	1.53–2.68	[30,39]
Complement factor D	P00746	CFD	3 (e, l)	3	1.19–2.81	[28,35]
Fibronectin	P02751	FN1	3 (e, m, l)	6	1.40–2.19	[28,32,34,35]
Insulin-like growth factor-binding protein 4	P22692	IGFBP4	3 (e, l)	3	1.19–30.1	[28,35]
Nidogen-1	P14543	NID1	3 (e, l)	3	1.36–2.18	[29,30]
Protein AMBP	P02760	AMBP	3 (e, l)	3	1.32–1.44	[28,42]
von Willebrand factor	P04275	VWF	3 (e, l)	3	1.52–1.60	[28,35,42]

## Data Availability

All relevant data are within the manuscript and its Supporting Information Files.

## Supplementary Materials

The following supporting information can be downloaded at https://www.mdpi.com/article/10.3390/life15050776/s1, Figure S1: Cohort size distributions for (A) PE, (B) Control, (C) Severe PE, and (D) FGR study groups, with median values (bold lines); Figure S2: Significantly enriched (FDR < 0.05) first-trimester PE protein associations: (A) GO biological processes; (B) Reactome pathways; Figure S3: Significantly enriched (FDR < 0.05) second-trimester PE protein associations: (A) GO biological processes; (B) KEGG pathways; (C) Reactome pathways; (D) Wikipathways; Figure S4: Significantly enriched (FDR < 0.05) third-trimester PE protein associations: (A) KEGG pathways; (B) GO biological processes; (C) Reactome pathways; (D) Wikipathways; Figure S5: Significantly enriched (FDR<0.05) third-trimester PE protein associations: (A) KEGG pathways; (B) GO biological processes; (C) Reactome pathways; (D) Wikipathways. Table S1: Studied cohort characteristics. Notation: X-Y = range; X(Y-Z) = mean(range); X ± Y=mean ± SD; X [Y;Z] = median [Q1;Q3]. Table S2: Study-specific protein alterations. Table S3: Consistently altered proteins (≥2 studies). Table S4: Counts of significantly altered proteins per study. Table S5: First trimester-specific protein alterations (≥2 studies) with matched directional changes. Table S6: First trimester-specific protein alterations (≥2 studies) with unmatched directional changes. Table S7: Second trimester-specific protein alterations (≥2 studies) with matched directional changes. Table S8: Second trimester-specific protein alterations (≥2 studies) with unmatched directional changes. Table S9: Third trimester-specific protein alterations (≥2 studies) with matched directional changes. Table S10: Third trimester-specific protein alterations (≥2 studies) with unmatched directional changes; Table S11: Pathway enrichment analysis of 1st trimester consistently altered proteins (≥2 studies); Table S12: Pathway enrichment analysis of 2nd trimester consistently altered proteins (≥2 studies); Table S13: Pathway enrichment analysis of 3rd trimester consistently altered proteins (≥2 studies).

## Abbreviations

The following abbreviations are used in this manuscript:

2-DE	Two-dimensional gel electrophoresis
2D-DIGE	Two-dimensional difference gel electrophoresis with fluorescent labeling
AUC	Area-under-the-curve
CXCL10	C-X-C motif chemokine ligand 10
ECM	Extracellular matrix
ELISA	Enzyme-linked immunosorbent assay
FGR	Fetal growth restriction
FIGO	International Federation of Gynecology and Obstetrics
FMF	Fetal Medicine Foundation
HELLP	Hemolysis, Elevated Liver Enzymes, Low Platelet Count
HPLC-MS/MS	High-performance liquid chromatography–tandem mass spectrometry
IQR	Inter-quartile range
IS	Isotope-labeled internal standards (tryptic peptides)
iTRAQ	Isobaric tags for relative and absolute quantitation
LC-MS/MS	Liquid chromatography -tandem mass spectrometry
MALDI-MS/MS	Matrix-assisted laser desorption/ionization–tandem mass spectrometry
MRM	Multiple reaction monitoring
NGS	Next-generation sequencing
NMR	Nuclear magnetic resonance
PAPP-A	Pregnancy-associated plasma protein-A
PCR	Polymerase chain reaction
PE	Preeclampsia
PlGF	Placental growth factor
SD	Standard deviation
SDS-PAGE	Polyacrylamide gel electrophoresis with sodium dodecyl sulfate
sFlt-1	Soluble fms-like tyrosine kinase-1
SOMAmer	Slow off-rate modified DNA aptamers
SRM	Selected reaction monitoring
SWATH	Sequential window acquisition of all theoretical mass spectra
TMT	Tandem mass tag

## References

[1] Lo J.O., Mission J.F., Caughey A.B. (2013). Hypertensive disease of pregnancy and maternal mortality. Curr. Opin. Obstet. Gynecol..

[2] Knight M., Bunch K., Felker A., Patel R., Kotnis R., Kenyon S., Kurinczuk J.J. (2023). MBRRACE—UK: Care Saving Lives, Improving Mothers’ Care Lessons learned to Inform Maternity Care from the UK and Ireland Confidential Enquiries into Maternal Deaths and Morbidity State of the Nation Surveillance Report 20.

[3] Boghossian N.S., Yeung E., Mendola P., Hinkle S.N., Laughon S.K., Zhang C., Albert P.S. (2014). Risk factors differ between recurrent and incident preeclampsia: A hospital-based cohort study. Ann. Epidemiol..

[4] NICE Guidelines Institute for Health and Care (2019). Hypertension in Pregnancy: Diagnosis and Management.

[5] Lisonkova S., Joseph K.S. (2013). Incidence of preeclampsia: Risk factors and outcomes associated with early- versus late-onset disease. Am. J. Obstet. Gynecol..

[6] Lisonkova S., Sabr Y., Mayer C., Young C., Skoll A., Joseph K.S. (2014). Maternal morbidity associated with early-onset and late-onset Preeclampsia. Obstet. Gynecol..

[7] Khan S., Siddique A.B., Jabeen S., Hossain A.T., Haider M.M., Zohora F.T., Rahman M.M., El Arifeen S., Rahman A.E., Jamil K. (2023). Preeclampsia and eclampsia-specific maternal mortality in Bangladesh: Levels, trends, timing, and care-seeking practices. J. Glob. Health.

[8] Melchiorre K., Sutherland G.R., Liberati M., Thilaganathan B. (2011). Preeclampsia is associated with persistent postpartum cardiovascular impairment. Hypertension.

[9] Melchiorre K., Sutherland G.R., Liberati M., Thilaganathan B. (2012). Maternal cardiovascular impairment in pregnancies complicated by severe fetal growth restriction. Hypertension.

[10] Jung E., Romero R., Yeo L., Gomez-Lopez N., Chaemsaithong P., Jaovisidha A., Gotsch F., Erez O. (2022). The etiology of preeclampsia. Am. J. Obstet. Gynecol..

[11] Staff A.C., Fjeldstad H.E., Fosheim I.K., Moe K., Turowski G., Johnsen G.M., Alnaes-Katjavivi P., Sugulle M. (2022). Failure of physiological transformation and spiral artery atherosis: Their roles in preeclampsia. Am. J. Obstet. Gynecol..

[12] Melchiorre K., Giorgione V., Thilaganathan B. (2022). The placenta and preeclampsia: Villain or victim?. Am. J. Obstet. Gynecol..

[13] American College of Obstetricians and Gynecologists (2018). Low-dose aspirin use during pregnancy. ACOG Committee Opinion No. 743. Obstet. Gynecol..

[14] Henderson J.T., Whitlock E.P., O’Connor E., Senger C.A., Thompson J.H., Rowland M.G. (2014). Low-dose aspirin for prevention of morbidity and mortality from preeclampsia: A systematic evidence review for the u.s. preventive services task force. Ann. Intern. Med..

[15] Youssef L., Testa L., Crovetto F., Crispi F. (2024). 10. Role of high dimensional technology in preeclampsia (omics in preeclampsia). Best Pract. Res. Clin. Obstet. Gynaecol..

[16] Stepan H., Herraiz I., Schlembach D., Verlohren S., Brennecke S., Chantraine F., Klein E., Lapaire O., Llurba E., Ramoni A. (2015). Implementation of the sFlt-1/PlGF ratio for prediction and diagnosis of pre-eclampsia in singleton pregnancy: Implications for clinical practice. Ultrasound Obstet. Gynecol..

[17] Zeisler H., Llurba E., Chantraine F., Vatish M., Staff A.C., Sennström M., Olovsson M., Brennecke S.P., Stepan H., Allegranza D. (2016). Predictive value of the sFlt-1:PlGF ratio in women with suspected preeclampsia. N. Engl. J. Med..

[18] Chaemsaithong P., Sahota D.S., Poon L.C. (2022). First trimester preeclampsia screening and prediction. Am. J. Obstet. Gynecol..

[19] Than N.G., Romero R., Posta M., Györffy D., Szalai G., Rossi S.W., Szilágyi A., Hupuczi P., Nagy S., Török O. (2024). Classification of preeclampsia according to molecular clusters with the goal of achieving personalized prevention. J. Reprod. Immunol..

[20] Ontario Health Technology Assessment Series (2022). First-Trimester Screening Program for the Risk of Pre-eclampsia Using a Multiple-Marker Algorithm: A Health Technology Assessment. Ont. Health Technol. Assess. Ser..

[21] Poon L.C., Shennan A., Hyett J.A., Kapur A., Hadar E., Divakar H., McAuliffe F., da Silva Costa F., von Dadelszen P., McIntyre H.D. (2019). The International Federation of Gynecology and Obstetrics (FIGO) initiative on pre-eclampsia: A pragmatic guide for first-trimester screening and prevention. Int. J. Gynaecol. Obstet. Off. Organ. Int. Fed. Gynaecol. Obstet..

[22] Page M.J., McKenzie J.E., Bossuyt P.M., Boutron I., Hoffmann T.C., Mulrow C.D., Shamseer L., Tetzlaff J.M., Akl E.A., Brennan S.E. (2021). The PRISMA 2020 statement: An updated guideline for reporting systematic reviews. BMJ.

[23] Wells G., Shea B., O’Connell D., Robertson J., Peterson J., Welch V., Losos M., Tugwell P. The Newcastle-Ottawa Scale (NOS) for Assessing the Quality of Nonrandomised Studies in Meta-Analyses. 2013, 21. https://www.ohri.ca/programs/clinical_epidemiology/nosgen.pdf.

[24] Szklarczyk D., Kirsch R., Koutrouli M., Nastou K., Mehryary F., Hachilif R., Gable A.L., Fang T., Doncheva N.T., Pyysalo S. (2023). The STRING database in 2023: Protein-protein association networks and functional enrichment analyses for any sequenced genome of interest. Nucleic Acids Res..

[25] Thomas P.D., Ebert D., Muruganujan A., Mushayahama T., Albou L.P., Mi H. (2022). PANTHER: Making genome-scale phylogenetics accessible to all. Protein Sci..

[26] Atkinson K.R., Blumenstein M., Black M.A., Wu S.H., Kasabov N., Taylor R.S., Cooper G.J.S., North R.A. (2009). An altered pattern of circulating apolipoprotein E3 isoforms is implicated in preeclampsia. J. Lipid Res..

[27] Beernink R.H.J., Zwertbroek E.F., Schuitemaker J.H.N., Cremers T.I.F.H., Scherjon S.A. (2022). First trimester serum biomarker discovery study for early onset, preterm onset and preeclampsia at term. Placenta.

[28] Chen H., Aneman I., Nikolic V., Karadzov Orlic N., Mikovic Z., Stefanovic M., Cakic Z., Jovanovic H., Town S.E.L., Padula M.P. (2022). Maternal plasma proteome profiling of biomarkers and pathogenic mechanisms of early-onset and late-onset preeclampsia. Sci. Rep..

[29] Degnes M.H.L., Westerberg A.C., Andresen I.J., Henriksen T., Roland M.C.P., Zucknick M., Michelsen T.M. (2024). Protein biomarker signatures of preeclampsia—A longitudinal 5000-multiplex proteomics study. Sci. Rep..

[30] Erez O., Romero R., Maymon E., Chaemsaithong P., Done B., Pacora P., Panaitescu B., Chaiworapongsa T., Hassan S.S., Tarca A.L. (2017). The prediction of late-onset preeclampsia: Results from a longitudinal proteomics study. PLoS ONE.

[31] Kolialexi A., Tsangaris G.T., Sifakis S., Gourgiotis D., Katsafadou A., Lykoudi A., Marmarinos A., Mavreli D., Pergialiotis V., Fexi D. (2017). Plasma biomarkers for the identification of women at risk for early-onset preeclampsia. Expert Rev. Proteom..

[32] Lim J.H., Lim J.M., Lee H.M., Lee H.J., Kwak D.W., Han Y.J., Kim M.Y., Jung S.H., Kim Y.R., Ryu H.M. (2024). Systematic Proteome Profiling of Maternal Plasma for Development of Preeclampsia Biomarkers. Mol. Cell Proteom..

[33] Liu L.Y., Yang T., Ji J., Wen Q., Morgan A.A., Jin B., Chen G., Lyell D.J., Stevenson D.K., Ling X.B. (2013). Integrating multiple “omics” analyses identifies serological protein biomarkers for preeclampsia. BMC Med..

[34] Lu Q., Liu C., Liu Y., Zhang N., Deng H., Zhang Z. (2016). Serum markers of pre-eclampsia identified on proteomics. J. Obstet. Gynaecol. Res..

[35] Odenkirk M.T., Stratton K.G., Gritsenko M.A., Bramer L.M., Webb-Robertson B.J.M., Bloodsworth K.J., Weitz K.K., Lipton A.K., Monroe M.E., Ash J.R. (2020). Unveiling molecular signatures of preeclampsia and gestational diabetes mellitus with multi-omics and innovative cheminformatics visualization tools. Mol. Omi..

[36] Shi X.-F., Zhang J.-L., Liu K., Wang L., Wang H.-P., Wu H.-Y. (2024). Detection of serum major histocompatibility complex I (HLA-1) and β2-microglobulin (β2M) in pre-eclampsia using isobaric tags for relative and absolute quantitation (iTRAQ). Int. J. Gynaecol. Obstet. Off. Organ. Int. Fed. Gynaecol. Obstet..

[37] Starodubtseva N., Tokareva A., Kononikhin A., Brzhozovskiy A., Bugrova A., Kukaev E., Muminova K., Nakhabina A., Frankevich V.E., Nikolaev E. (2024). First-Trimester Preeclampsia-Induced Disturbance in Maternal Blood Serum Proteome: A Pilot Study. Int. J. Mol. Sci..

[38] Than N.G., Romero R., Tarca A.L., Kekesi K.A., Xu Y., Xu Z., Juhasz K., Bhatti G., Leavitt R.J., Gelencser Z. (2018). Integrated systems biology approach identifies novel maternal and placental pathways of preeclampsia. Front. Immunol..

[39] Than N.G., Posta M., Györffy D., Orosz L., Orosz G., Rossi S.W., Ambrus-Aikelin G., Szilágyi A., Nagy S., Hupuczi P. (2022). Early pathways, biomarkers, and four distinct molecular subclasses of preeclampsia: The intersection of clinical, pathological, and high-dimensional biology studies. Placenta.

[40] Uchida Y., Higuchi T., Shirota M., Kagami S., Saigusa D., Koshiba S., Yasuda J., Tamiya G., Kuriyama S., Kinoshita K. (2021). Identification and validation of combination plasma biomarker of afamin, fibronectin and sex hormone-binding globulin to predict pre-eclampsia. Biol. Pharm. Bull..

[41] Wang X., Yip K.C., He A., Tang J., Liu S., Yan R., Zhang Q., Li R. (2022). Plasma Olink Proteomics Identifies CCL20 as a Novel Predictive and Diagnostic Inflammatory Marker for Preeclampsia. J. Proteome Res..

[42] Youssef L., Miranda J., Blasco M., Paules C., Crovetto F., Palomo M., Torramade-Moix S., García-Calderó H., Tura-Ceide O., Dantas A.P. (2021). Complement and coagulation cascades activation is the main pathophysiological pathway in early-onset severe preeclampsia revealed by maternal proteomics. Sci. Rep..

[43] Dimitriadis E., Rolnik D.L., Zhou W., Estrada-Gutierrez G., Koga K., Francisco R.P.V., Whitehead C., Hyett J., da Silva Costa F., Nicolaides K. (2023). Pre-eclampsia. Nat. Rev. Dis. Prim..

[44] Burwick R.M., Feinberg B.B. (2022). Complement activation and regulation in preeclampsia and hemolysis, elevated liver enzymes, and low platelet count syndrome. Am. J. Obstet. Gynecol..

[45] Girardi G., Lingo J.J., Fleming S.D., Regal J.F. (2020). Essential Role of Complement in Pregnancy: From Implantation to Parturition and Beyond. Front. Immunol..

[46] Matsuyama T., Tomimatsu T., Mimura K., Yagi K., Kawanishi Y., Kakigano A., Nakamura H., Endo M., Kimura T. (2021). Complement activation by an angiogenic imbalance leads to systemic vascular endothelial dysfunction: A new proposal for the pathophysiology of preeclampsia. J. Reprod. Immunol..

[47] Fischetti F., Tedesco F. (2006). Cross-talk between the complement system and endothelial cells in physiologic conditions and in vascular diseases. Autoimmunity.

[48] Than N.G., Romero R., Györffy D., Posta M., Bhatti G., Done B., Chaemsaithong P., Jung E., Suksai M., Gotsch F. (2023). Molecular subclasses of preeclampsia characterized by a longitudinal maternal proteomics study: Distinct biomarkers, disease pathways and options for prevention. J. Perinat. Med..

[49] Balduit A., Agostinis C., Mangogna A., Zito G., Stampalija T., Ricci G., Bulla R. (2024). Systematic review of the complement components as potential biomarkers of pre-eclampsia: Pitfalls and opportunities. Front. Immunol..

[50] Regal J.F., Burwick R.M., Fleming S.D. (2017). The Complement System and Preeclampsia. Curr. Hypertens. Rep..

[51] Pierik E., Prins J.R., van Goor H., Dekker G.A., Daha M.R., Seelen M.A.J., Scherjon S.A. (2020). Dysregulation of Complement Activation and Placental Dysfunction: A Potential Target to Treat Preeclampsia?. Front. Immunol..

[52] Welsh A., National Collaborating Centre for Women’s and Children’s Health (2010). Hypertension in Pregnancy: The Management of Hypertensive Disorders During Pregnancy Hypertension in Pregnancy the Management of Hypertensive Disorders.

[53] Ducat A., Vargas A., Doridot L., Bagattin A., Lerner J., Vilotte J.L., Buffat C., Pontoglio M., Miralles F., Vaiman D. (2019). Low-dose aspirin protective effects are correlated with deregulation of HNF factor expression in the preeclamptic placentas from mice and humans. Cell Death Discov..

[54] Nguyen T.P.H., Patrick C.J., Parry L.J., Familari M. (2019). Using proteomics to advance the search for potential biomarkers for preeclampsia: A systematic review and meta-analysis. PLoS ONE.

[55] Ricklin D., Lambris J.D. (2013). Complement in Immune and Inflammatory Disorders: Pathophysiological Mechanisms. J. Immunol..

[56] Levi M., Van Der Poll T. (2010). Inflammation and coagulation. Crit. Care Med..

[57] Huang J., Li X., Shi X., Zhu M., Wang J., Huang S., Huang X., Wang H., Li L., Deng H. (2019). Platelet integrin αiIbβ3: Signal transduction, regulation, and its therapeutic targeting. J. Hematol. Oncol..

[58] Tarca A.L., Romero R., Benshalom-Tirosh N., Than N.G., Gudicha D.W., Done B., Pacora P., Chaiworapongsa T., Panaitescu B., Tirosh D. (2019). The prediction of early preeclampsia: Results from a longitudinal proteomics study. PLoS ONE.

[59] Bahabayi A., Yang N., Xu T., Xue Y., Ma L., Gu X., Wang Y., Jia K. (2022). Expression of Matrix Metalloproteinase-2,-7,-9 in Serum during Pregnancy in Patients with Pre-Eclampsia: A Prospective Study. Int. J. Environ. Res. Public Health.

[60] Jia Y., Lu W., Xie H., Sheng Y., Wang L., Lv W., Ling L., Dong J., Jia X., Wu S. (2024). Upregulation of Siglec-6 induces mitochondrial dysfunction by promoting GPR20 expression in early-onset preeclampsia. J. Transl. Med..

[61] Rumer K.K., Uyenishi J., Hoffman M.C., Fisher B.M., Winn V.D. (2013). Siglec-6 expression is increased in placentas from pregnancies complicated by preterm preeclampsia. Reprod. Sci..

[62] Maynard S.E., Min J., Merchan J., Lim K., Li J., Mondal S., Libermann T.A., Morgan J.P., Sellke F.W., Stillman I.E. (2003). Excess placental soluble fms-like tyrosine kinase 1 (sFlt1) may contribute to endothelial dysfunction, hypertension, and proteinuria in preeclampsia. J. Clin. Investig..

[63] Maragoudakis M.E. (2003). Angiogenesis in health and disease. Nat. Med..

[64] Bellamy L., Casas J.P., Hingorani A.D., Williams D.J. (2007). Pre-eclampsia and risk of cardiovascular disease and cancer in later life: Systematic review and meta-analysis. Br. Med. J..

[65] Wu P., Haththotuwa R., Kwok C.S., Babu A., Kotronias R.A., Rushton C., Zaman A., Fryer A.A., Kadam U., Chew-Graham C.A. (2017). Preeclampsia and future cardiovascular health. Circ. Cardiovasc. Qual. Outcomes.

[66] Lisowska M., Pietrucha T., Sakowicz A. (2018). Preeclampsia and Related Cardiovascular Risk: Common Genetic Background. Curr. Hypertens. Rep..

[67] Jacobsen D.P., Røysland R., Strand H., Moe K., Sugulle M., Omland T., Staff A.C. (2022). Circulating cardiovascular biomarkers during and after preeclampsia: Crosstalk with placental function?. Pregnancy Hypertens..

[68] O’Brien T.E., Ray J.G., Chan W.-S. (2003). Maternal Body Mass Index and The Risk of Preeclampsia. A Systematic overview. Epidemiology.

[69] Sierra-Laguado J., García R.G., Celedón J., Arenas-Mantilla M., Pradilla L.P., Camacho P.A., López-Jaramillo P. (2007). Determination of Insulin Resistance Using the Homeostatic Model Assessment (HOMA) and its Relation With the Risk of Developing Pregnancy-Induced Hypertension. Am. J. Hypertens..

[70] García R.G., Celedón J., Sierra-Laguado J., Alarcón M.A., Luengas C., Silva F., Arenas-Mantilla M., López-Jaramillo P. (2007). Raised C-Reactive Protein and Impaired Flow-Mediated Vasodilation Precede the Development of Preeclampsia. Am. J. Hypertens..

[71] Lopez-Jaramillo P., Barajas J., Rueda-Quijano S.M., Lopez-Lopez C., Felix C. (2018). Obesity and Preeclampsia: Common Pathophysiological Mechanisms. Front. Physiol..

[72] López-Jaramillo P., Terán E., Ringqvist A., Moya W., Rivera J., Berrazueta J.R. (1997). P-375—Oxidised low-density lipoproteins and nitric oxide during normal pregnancy and preeclampsia. Jpn. J. Pharmacol..

[73] Reyes L.M., García R.G., Ruiz S.L., Broadhurst D., Aroca G., Davidge S.T., López-Jaramillo P. (2012). Angiogenic imbalance and plasma lipid alterations in women with preeclampsia from a developing country. Growth Factors.

[74] Reyes L.M., García R.G., Ruiz S.L., Camacho P.A., Ospina M.B., Aroca G., Accini J.L., López-Jaramillo P. (2012). Risk factors for preeclampsia in women from Colombia: A case-control study. PLoS ONE.

[75] Hubel C.A., McLaughlin M.K., Evans R.W., Hauth B.A., Sims C.J., Roberts J.M. (1996). Fasting serum triglycerides, free fatty acids, and malondialdehyde are increased in preeclampsia, are positively correlated, and decrease within 48 hours post partum. Am. J. Obstet. Gynecol..

[76] Staff A.C., Dechend R., Pijnenborg R. (2010). Learning from the placenta: Acute atherosis and vascular remodeling in preeclampsia-novel aspects for atherosclerosis and future cardiovascular health. Hypertension.

[77] Von Eckardstein A., Nordestgaard B.G., Catapano A.L., Remaley A.T. (2023). High-density lipoprotein revisited: Biological functions and clinical relevance. Eur. Heart J..

[78] Brites F., Martin M., Guillas I., Kontush A. (2017). Antioxidative activity of high-density lipoprotein (HDL): Mechanistic insights into potential clinical bene fi t. BBA Clin..

[79] Shah A.S., Tan L., Long J.L., Davidson W.S. (2013). Proteomic diversity of high density lipoproteins: Our emerging understanding of its importance in lipid transport and beyond. J. Lipid Res..

[80] Stadler J.T., Scharnagl H., Wadsack C., Marsche G. (2023). Preeclampsia Affects Lipid Metabolism and HDL Function in Mothers and Their Offspring. Antioxidants.

[81] Bellos I., Papantoniou N., Pergialiotis V. (2018). Serum ceruloplasmin levels in preeclampsia: A meta-analysis. J. Matern. Neonatal Med..

[82] Sak S., Barut M., Çelik H., Incebiyik A., Ağaçayak E., Uyanikoglu H., Kirmit A., Sak M. (2020). Copper and ceruloplasmin levels are closely related to the severity of preeclampsia. J. Matern. Neonatal Med..

[83] Gaillard R., Arends L.R., Steegers E.A.P., Hofman A., Jaddoe V.W.V. (2013). Second-and third-trimester placental hemodynamics and the risks of pregnancy complications. Am. J. Epidemiol..

[84] Myatt L., Cui X. (2004). Oxidative stress in the placenta. Histochem. Cell Biol..

[85] Raijmakers M.T.M., Dechend R., Poston L. (2004). Oxidative stress and preeclampsia: Rationale for antioxidant clinical trials. Hypertension.

[86] Mao Y., Schwarzbauer J.E. (2005). Fibronectin fibrillogenesis, a cell-mediated matrix assembly process. Matrix Biol..

[87] Peng Z., Hao M., Tong H., Yang H., Huang B., Zhang Z., Luo K.Q. (2022). The interactions between integrin α5β1 of liver cancer cells and fibronectin of fibroblasts promote tumor growth and angiogenesis. Int. J. Biol. Sci..

[88] Uhlén M., Fagerberg L., Hallström B.M., Lindskog C., Oksvold P., Mardinoglu A., Sivertsson Å., Kampf C., Sjöstedt E., Asplund A. (2015). Tissue-based map of the human proteome. Science.

[89] Goldman-Wohl D., Yagel S. (2002). Regulation of trophoblast invasion: From normal implantation to pre-eclampsia. Mol. Cell. Endocrinol..

[90] Chavarría M.E., Lara-González L., González-Gleason A., Sojo I., Reyes A. (2002). Maternal plasma cellular fibronectin concentrations in normal and preeclamptic pregnancies: A longitudinal study for early prediction of preeclampsia. Am. J. Obstet. Gynecol..

[91] Rasanen J., Girsen A., Lu X., Lapidus J.A., Standley M., Reddy A., Dasari S., Thomas A., Jacob T., Pouta A. (2010). Comprehensive maternal serum proteomic profiles of preclinical and clinical preeclampsia. J. Proteome Res..

[92] Rasanen J., Quinn M.J., Laurie A., Bean E., Roberts C.T., Nagalla S.R., Gravett M.G. (2015). Maternal serum glycosylated fibronectin as a point-of-care biomarker for assessment of preeclampsia. Am. J. Obstet. Gynecol..

[93] Moungmaithong S., Wang X., Lau C.S.L., Tse A.W.T., Lee N.M.W., Leung H.H.Y., Poon L.C., Sahota D.S. (2023). Glycosylated fibronectin improves first-trimester prediction of pre-eclampsia. Ultrasound Obstet. Gynecol..

[94] Rifai N., Gillette M.A., Carr S.A. (2006). Protein biomarker discovery and validation: The long and uncertain path to clinical utility. Nat. Biotechnol..

[95] Brandwijk R.J.M.G.E., Michels M.A.H.M., van Rossum M., de Nooijer A.H., Nilsson P.H., de Bruin W.C.C., Toonen E.J.M. (2022). Pitfalls in complement analysis: A systematic literature review of assessing complement activation. Front. Immunol..

[96] Smejkal G.B. (2012). Proteomics Sample Preparation, Preservation, and Fractionation. Int. J. Proteom..

[97] Klont F., Bras L., Wolters J.C., Ongay S., Bischoff R., Halmos G.B., Horvatovich P. (2018). Assessment of Sample Preparation Bias in Mass Spectrometry-Based Proteomics. Anal. Chem..

[98] Guryča V., Roeder D., Piraino P., Lamerz J., Ducret A., Langen H., Cutler P. (2014). Automated sample preparation platform for mass spectrometry-based plasma proteomics and biomarker discovery. Biology.

[99] Yang S., McGookey M., Wang Y., Cataland S.R., Wu H.M. (2015). Effect of blood sampling, processing, and storage on the measurement of complement activation biomarkers. Am. J. Clin. Pathol..

[100] Hassis M.E., Niles R.K., Braten M.N., Albertolle M.E., Ewa Witkowska H., Hubel C.A., Fisher S.J., Williams K.E. (2015). Evaluating the effects of preanalytical variables on the stability of the human plasma proteome. Anal. Biochem..

[101] Han L., Holland O.J., Da Silva Costa F., Perkins A. (2023). V Potential biomarkers for late-onset and term preeclampsia: A scoping review. Front. Physiol..

[102] Leavey K., Grynspan D., Cox B.J. (2019). Both “canonical” and “immunological” preeclampsia subtypes demonstrate changes in placental immune cell composition. Placenta.

[103] Vasapollo B., Zullino S., Novelli G.P., Farsetti D., Ottanelli S., Clemenza S., Micaglio M., Ferrazzi E., Di Martino D.D., Ghi T. (2024). Maternal Hemodynamics from Preconception to Delivery: Research and Potential Diagnostic and Therapeutic Implications: Position Statement by Italian Association of Preeclampsia and Italian Society of Perinatal Medicine. Am. J. Perinatol..

