# Supplementary material for: Proteome-Based Maternal Plasma and Serum Biomarkers for Preeclampsia: A Systematic Review and Meta-Analysis
Source: Life (Basel). 2025 May 13;15(5):776. doi: 10.3390/life15050776 (PMC12113278; doi:10.3390/life15050776)
Supplement: Supplementary file 1 [file life-15-00776-s001.zip › Supplementary_Figures.pdf]

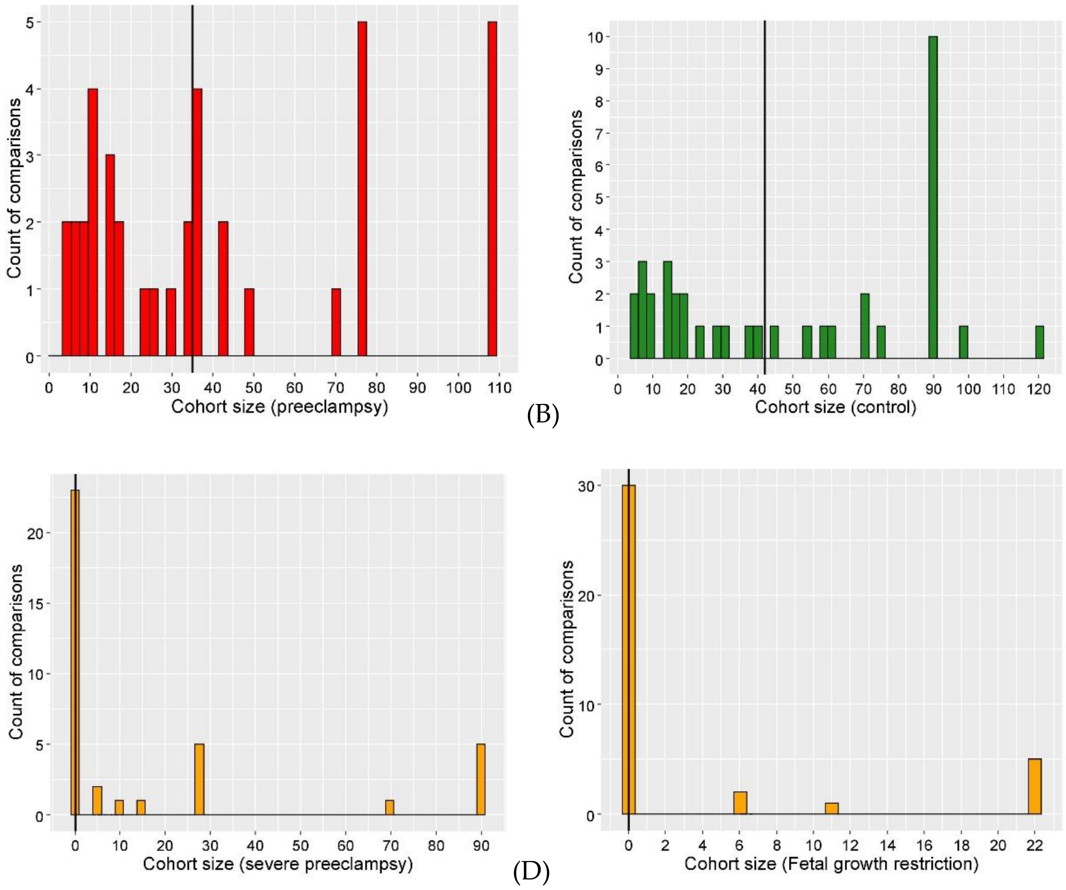

(A) (B) (C) (D)

Figure S1. Cohort size distributions for (A) PE, (B) Control, (C) Severe PE, and (D) FGR study groups, with median values (bold lines).

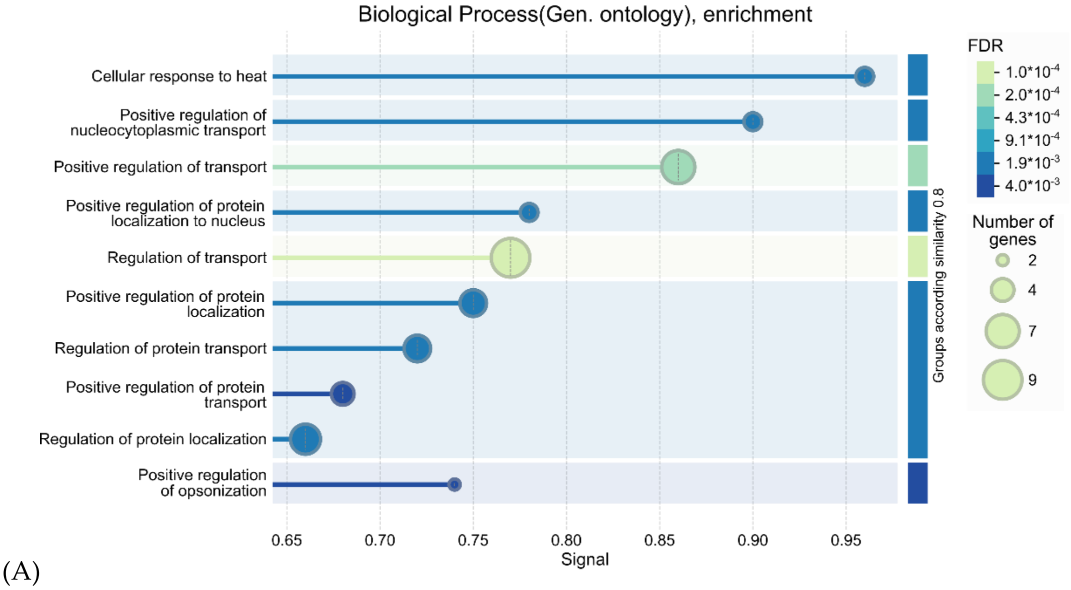

(A)

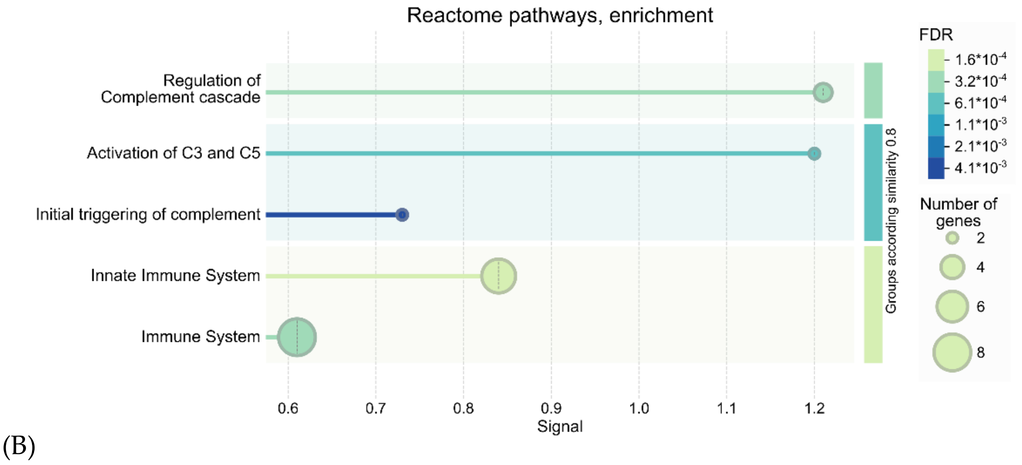

(B)

Figure S2. Significantly enriched (FDR<0.05) first-trimester PE protein associations: (A) GO biological processes; (B) Reactome pathways.

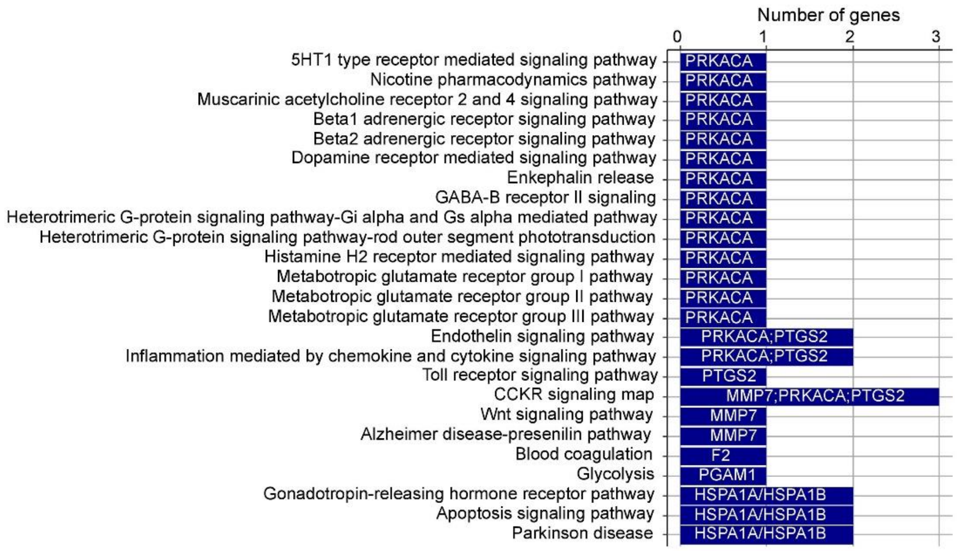

(A)

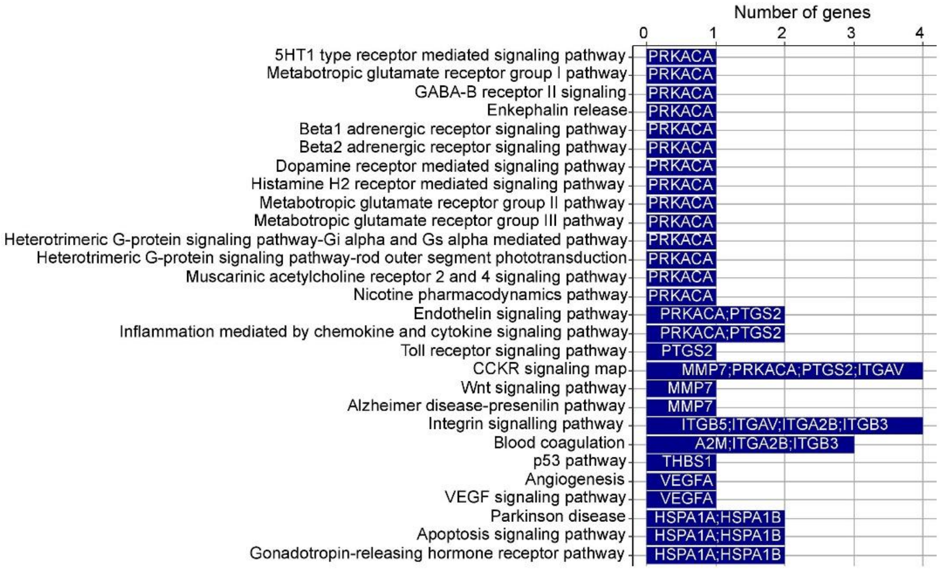

(B)

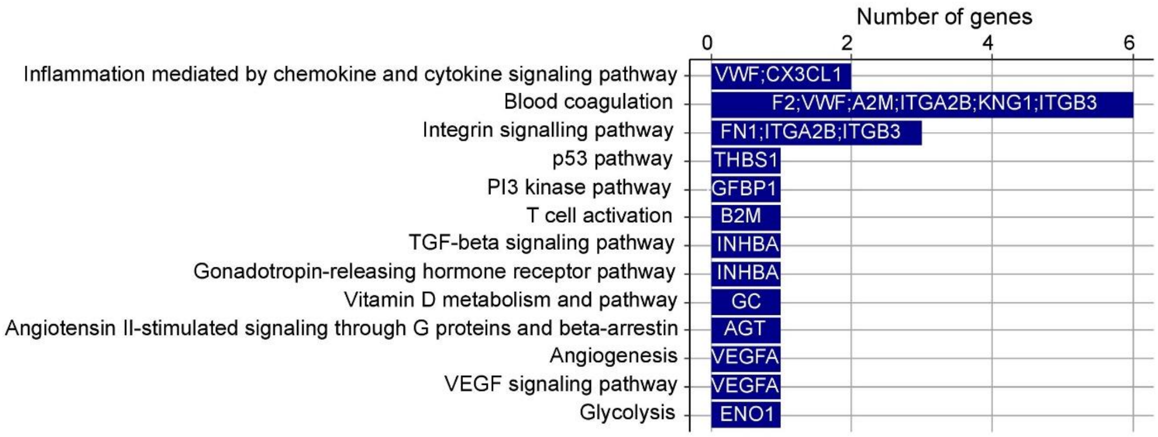

(C)

Figure S3. Pathway enrichment analysis of genes encoding differentially expressed proteins ($p<0.05$, replicated in ≥2 cohorts) stratified by trimester: (A) First, (B) Second, (C) Third.

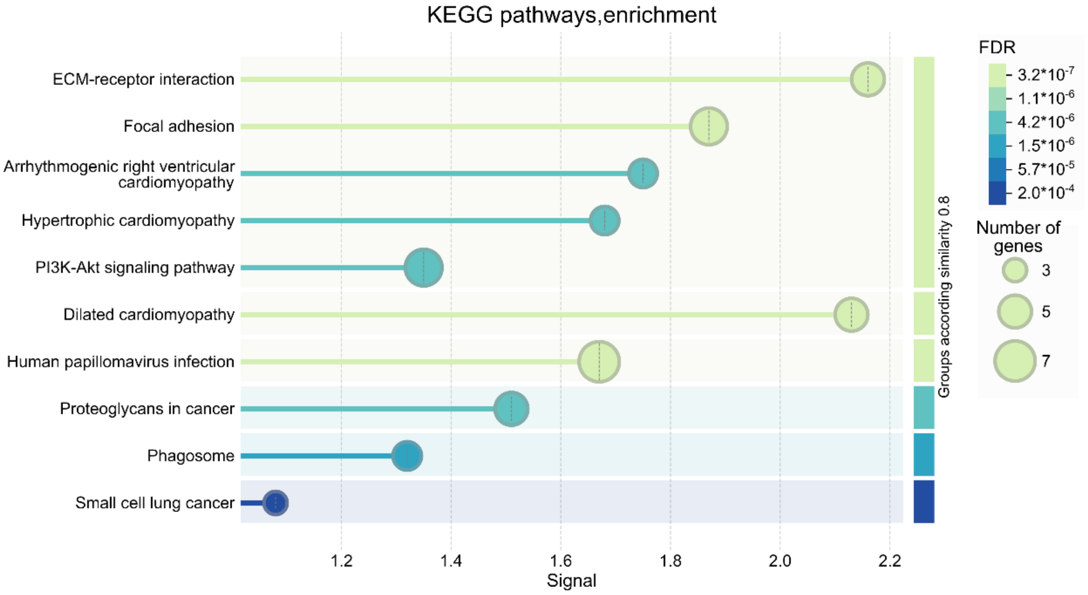

(A)

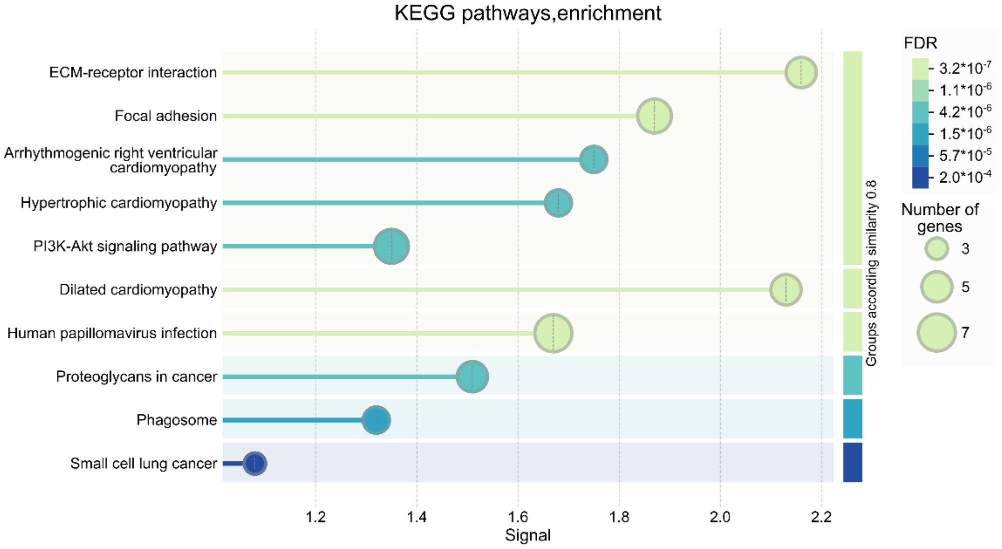

(B)

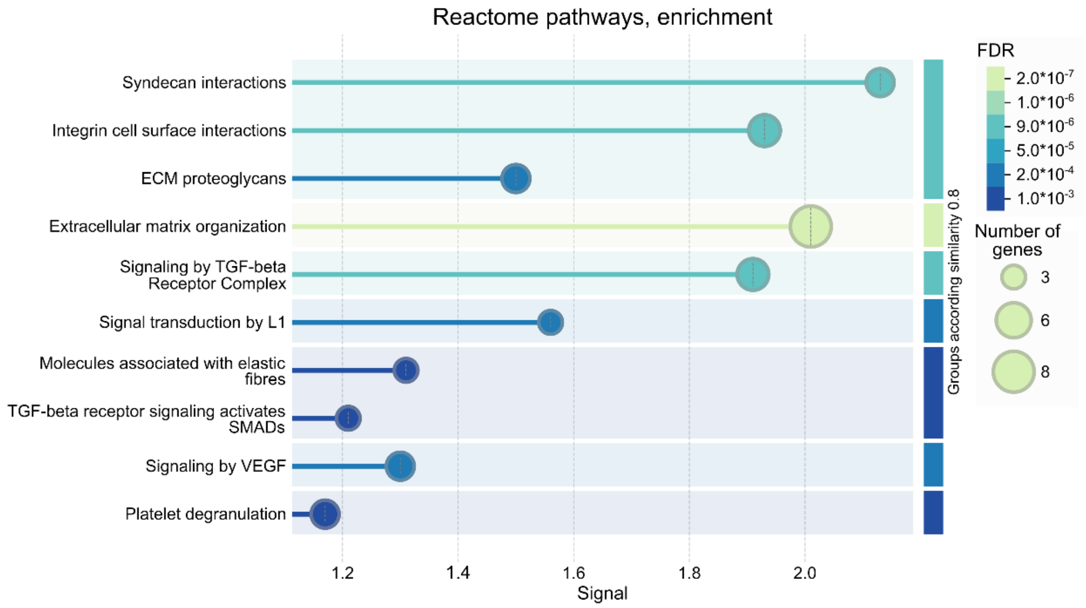

(C)

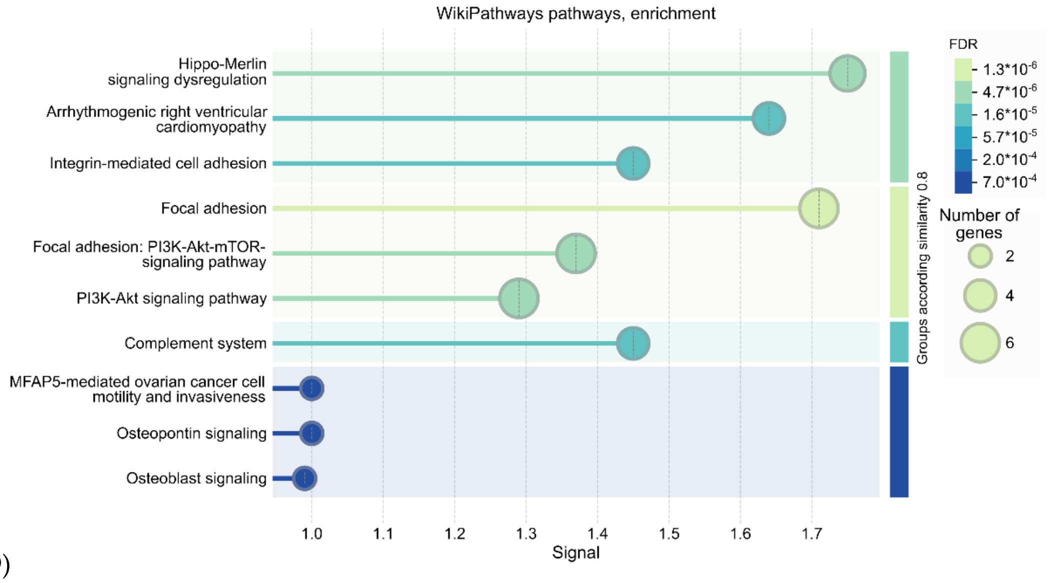

(D)

Figure S4. Significantly enriched (FDR<0.05) second-trimester PE protein associations: (A) GO biological processes; (B) KEGG pathways; (C) Reactome pathways; (D) Wikipathways.

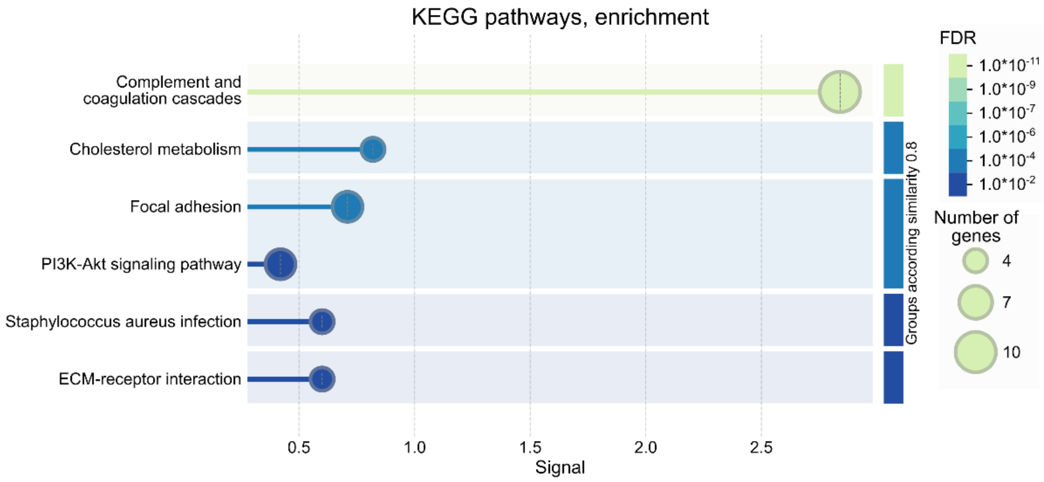
KEGG pathways, enrichment
Complement and coagulation cascades
Cholesterol metabolism
Focal adhesion
PI3K-Akt signaling pathway
Staphylococcus aureus infection
ECM-receptor interaction
0.5
1.0
1.5
2.0
2.5
Signal
Groups according similarity 0.8
FDR
$1.0*10^{-11}$
$1.0*10^{-9}$
$1.0*10^{-7}$
$1.0*10^{-6}$
$1.0*10^{-4}$
$1.0*10^{-2}$
Number of genes
4
7
10

(A)

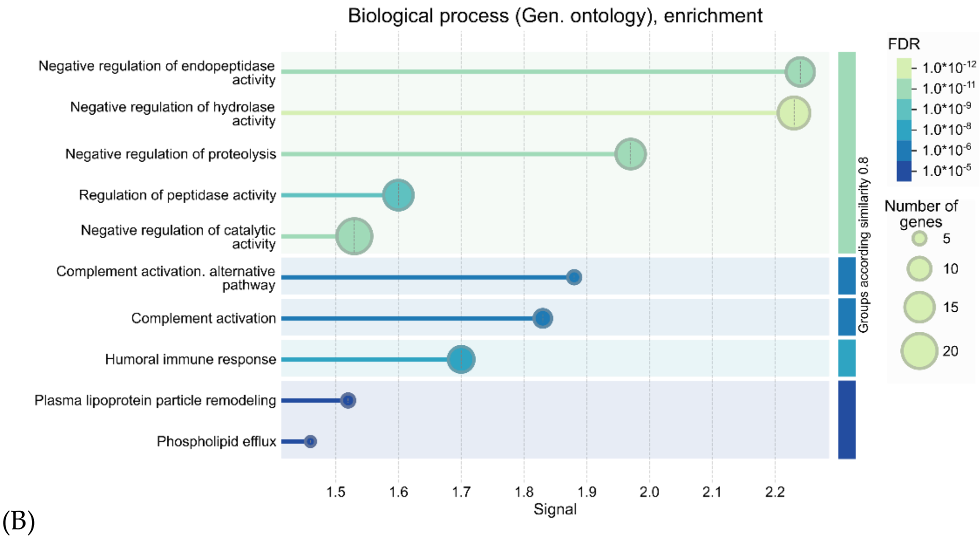
Biological process (Gen. ontology), enrichment
Negative regulation of endopeptidase activity
Negative regulation of hydrolase activity
Negative regulation of proteolysis
Regulation of peptidase activity
Negative regulation of catalytic activity
Complement activation. alternative pathway
Complement activation
Humoral immune response
Plasma lipoprotein particle remodeling
Phospholipid efflux
1.5
1.6
1.7
1.8
1.9
2.0
2.1
2.2
Signal
Groups according similarity 0.8
FDR
$1.0*10^{-12}$
$1.0*10^{-11}$
$1.0*10^{-9}$
$1.0*10^{-8}$
$1.0*10^{-6}$
$1.0*10^{-5}$
Number of genes
5
10
15
20

(B)

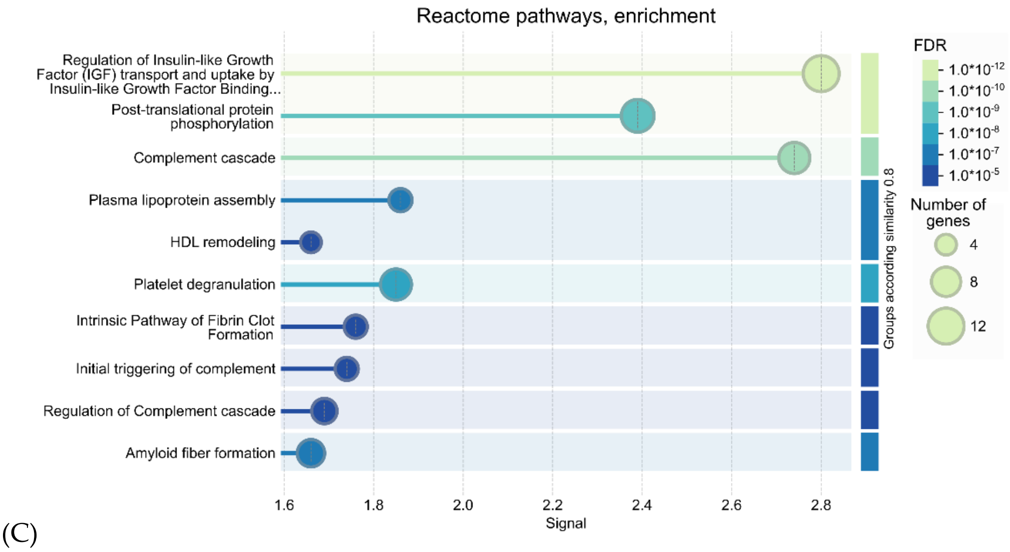
Reactome pathways, enrichment
Regulation of Insulin-like Growth Factor (IGF) transport and uptake by Insulin-like Growth Factor Binding...
Post-translational protein phosphorylation
Complement cascade
Plasma lipoprotein assembly
HDL remodeling
Platelet degranulation
Intrinsic Pathway of Fibrin Clot Formation
Initial triggering of complement
Regulation of Complement cascade
Amyloid fiber formation
1.6
1.8
2.0
2.2
2.4
2.6
2.8
Signal
Groups according similarity 0.8
FDR
$1.0*10^{-12}$
$1.0*10^{-10}$
$1.0*10^{-9}$
$1.0*10^{-8}$
$1.0*10^{-7}$
$1.0*10^{-5}$
Number of genes
4
8
12

(C)

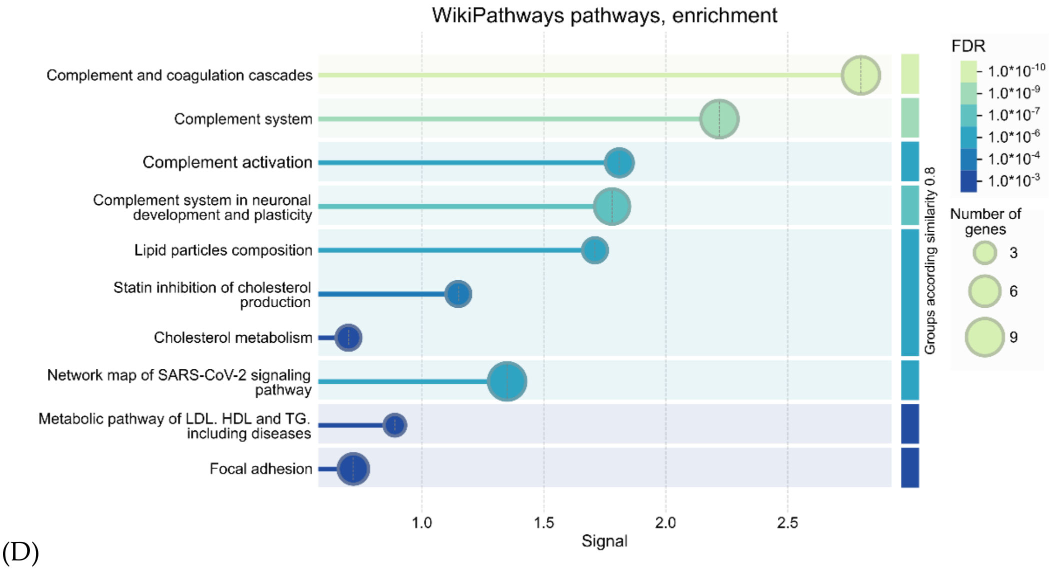

(D)

Figure S5. Significantly enriched (FDR<0.05) third-trimester PE protein associations: (A) KEGG pathways; (B) GO biological processes; (C) Reactome pathways; (D) Wikipathways.
